# Human hair keratin modification enhances the biocompatibility, bioactivity and osteogenic property of BCP/PMMA bone cement

**DOI:** 10.1093/rb/rbag157

**Published:** 2026-07-22

**Authors:** Zhigang Li, Xinyu Zhao, Yuchen Cao, Yadi Niu, Haifeng Wang, Xiaoyan Gong, Peng Li, Yen Wei, Kangmin Niu

**Affiliations:** School of Materials Science and Engineering, University of Science and Technology, Beijing 100083, China; The Key Laboratory of Bioorganic Phosphorus Chemistry & Chemical Biology (Ministry of Education), Department of Chemistry, Tsinghua University, Beijing 100084, China; School of Materials Science and Engineering, University of Science and Technology, Beijing 100083, China; School of Materials Science and Engineering, University of Science and Technology, Beijing 100083, China; Department of Neurosurgery, Beijing Hospital, Beijing 100730, China; Beijing ORGEN Biotechnology Co. Ltd, Beijing 100000, China; Hand Surgery Center, Xi’an Honghui Hospital, Xi’an Jiaotong University Health Science Center, Xi’an 710054, Shaanxi, China; The Key Laboratory of Bioorganic Phosphorus Chemistry & Chemical Biology (Ministry of Education), Department of Chemistry, Tsinghua University, Beijing 100084, China; School of Materials Science and Engineering, University of Science and Technology, Beijing 100083, China

**Keywords:** human hair keratin, biphasic calcium phosphate, PMMA bone cement, osteogenesis, bone repair

## Abstract

Biphasic calcium phosphate (BCP)/poly(methyl methacrylate) (PMMA) bone cement is an effective fixation material for bone tissue regeneration but has drawbacks, including poor hydrophilicity and low biological activity. To overcome these drawbacks, we present a novel human hair keratin-modified BCP/PMMA (KBP) composite. In this study, the influence of human hair keratin on the setting properties, mechanical strength, hydrophilicity, *in vitro* bioactivity and *in vivo* biocompatibility of KBP bone cement was investigated. Results show that incorporation of human hair-derived keratin improves the setting properties and hydrophilicity of bone cement, as reflected by a 16% reduction in contact angle, 80% increase in water absorption, 21% increase in porosity and 6% decrease in maximum setting temperature. The modification also improved mesenchymal stem cell viability by 15% and enhanced osteogenic differentiation. The keratin-based composite markedly promoted bone repair in a Sprague–Dawley rat bone defect model, with bone mineral density elevated by 17% and bone volume fraction increased by 15%. *In vivo* studies further demonstrated that KBP is nontoxic, nonpyrogenic and nonsensitizing, underscoring its potential as a safe and effective alternative for bone tissue repair. In summary, human hair keratin-modified bone cement is a promising candidate for orthopedic clinical applications.

## Graphical Abstract

**Graphical Abstract:**
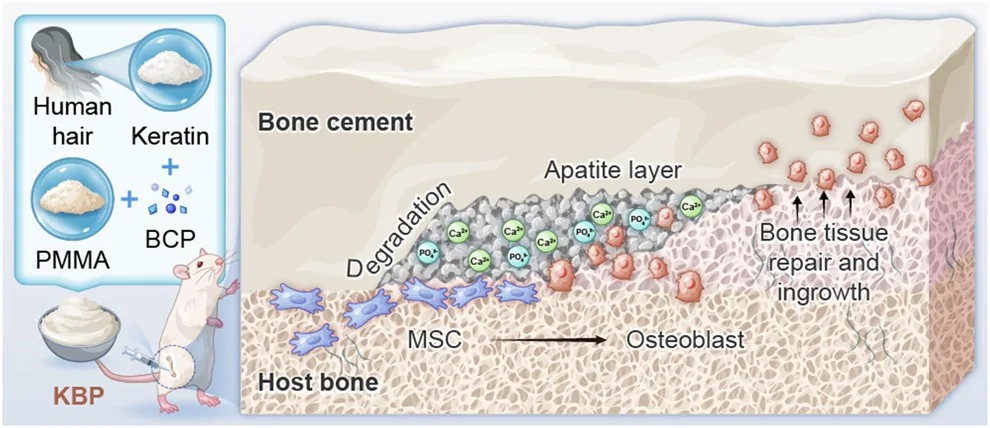

## Introduction

Poly(methyl methacrylate) (PMMA) bone cement has been a fundamental fixation agent in orthopedic surgery for more than 50 years, supporting procedures such as joint replacement, spinal fusion and fracture stabilization [1]. Renowned for its strong mechanical performance, ease of manipulation and reliable integration at the bone-cement interface, it is recognized as a highly effective biomaterial for bone repair [2]. Despite these advantages, PMMA carries limitations, including inadequate long-term biocompatibility, a lack of bioactivity, low porosity and the risk of thermal injury to surrounding tissues due to its exothermic curing reaction [3]. Moreover, because PMMA is inert and nondegradable, it often impedes the ingrowth of host bone tissue, which can substantially hinder the adhesion and proliferation of bone cells [4]. Consequently, these issues underscore the need to improve the structural and biological properties of PMMA bone cement to achieve better clinical outcomes.

Researchers have explored various additives to augment the overall functionality of PMMA bone cement, including linoleic acid [5], graphene oxide [6], carbon nanotube [7], hydroxyapatite (HA) [8, 9] and tricalcium phosphate (TCP) [10]. Among these, biphasic calcium phosphate (BCP), a combination of HA and β-TCP, has attracted particular interest due to the synergistic effects of the mechanical stability and bioactivity of HA and the resorbability and biodegradability of β-TCP [11]. Composites incorporating BCP have shown better biocompatibility and tunable degradation behavior, outperforming materials that use HA or β-TCP alone [12]. The integration of BCP into PMMA bone cement facilitates the formation of HA on its surface during degradation, which enhances bonding at the bone–cement interface and reduces the risk of implant loosening [2]. Furthermore, BCP has been found to support the adhesion, proliferation and differentiation of bone marrow mesenchymal stem cells (MSCs) [13]. Nevertheless, the osteogenic and chondrogenic differentiation capacity of BCP/PMMA bone cement remains limited when compared with biomaterials containing active biological cues, indicating the need for further biofunctional modification.

Keratin, a major structural protein found in hair, wool, feathers and nails, is an animal-derived osteoconductive material that can be obtained without sacrificing animals [14]. As a naturally biodegradable biomaterial, it supports somatic cell interactions and displays excellent biocompatibility, typically not triggering an immune response post-transplantation [15]. Specifically, keratin derived from a patient’s own hair has the potential to minimize the risk of immune rejection due to its autologous origin, compared with keratin from heterologous sources such as wool or feathers. Beyond its low immunogenicity, keratin also possesses abundant natural cell-binding motifs that substantially enhance cellular adhesion and proliferation, thereby promoting cell growth and differentiation [16, 17]. The interaction between keratin and cell-surface receptors, including integrins, facilitates the formation of focal adhesions [18], which is a key step in signaling pathways that regulate cell survival and cell-cycle progression. Owing to these bioactive features, keratin-based biomaterials have been widely investigated for tissue repair and regeneration [19, 20]. Wool-derived keratin scaffolds have been shown to promote cell adhesion and proliferation [14], while the incorporation of HA into such scaffolds can influence preosteoblast differentiation [21]. Likewise, electrospun feather keratin fibers support the growth and chondrogenic differentiation of adipose-derived MSCs [22]. These studies indicate that keratin is a promising component for improving the biological performance of bone- and cartilage-related biomaterials [23]. Notably, human hair keratin has also been demonstrated to modulate mesenchymal stem cell behavior and support osteogenic differentiation [24, 25]. However, its incorporation into BCP/PMMA bone cement and its combined effects on cement handling behavior, exothermic curing, mechanical integrity, cytocompatibility, osteogenic/chondrogenic differentiation and *in vivo* bone repair remain insufficiently explored. Therefore, clarifying how human hair keratin modulates the physicochemical and biological performance of BCP/PMMA cement is important for the development of bioactive PMMA-based bone repair materials.

**Figure 1:**
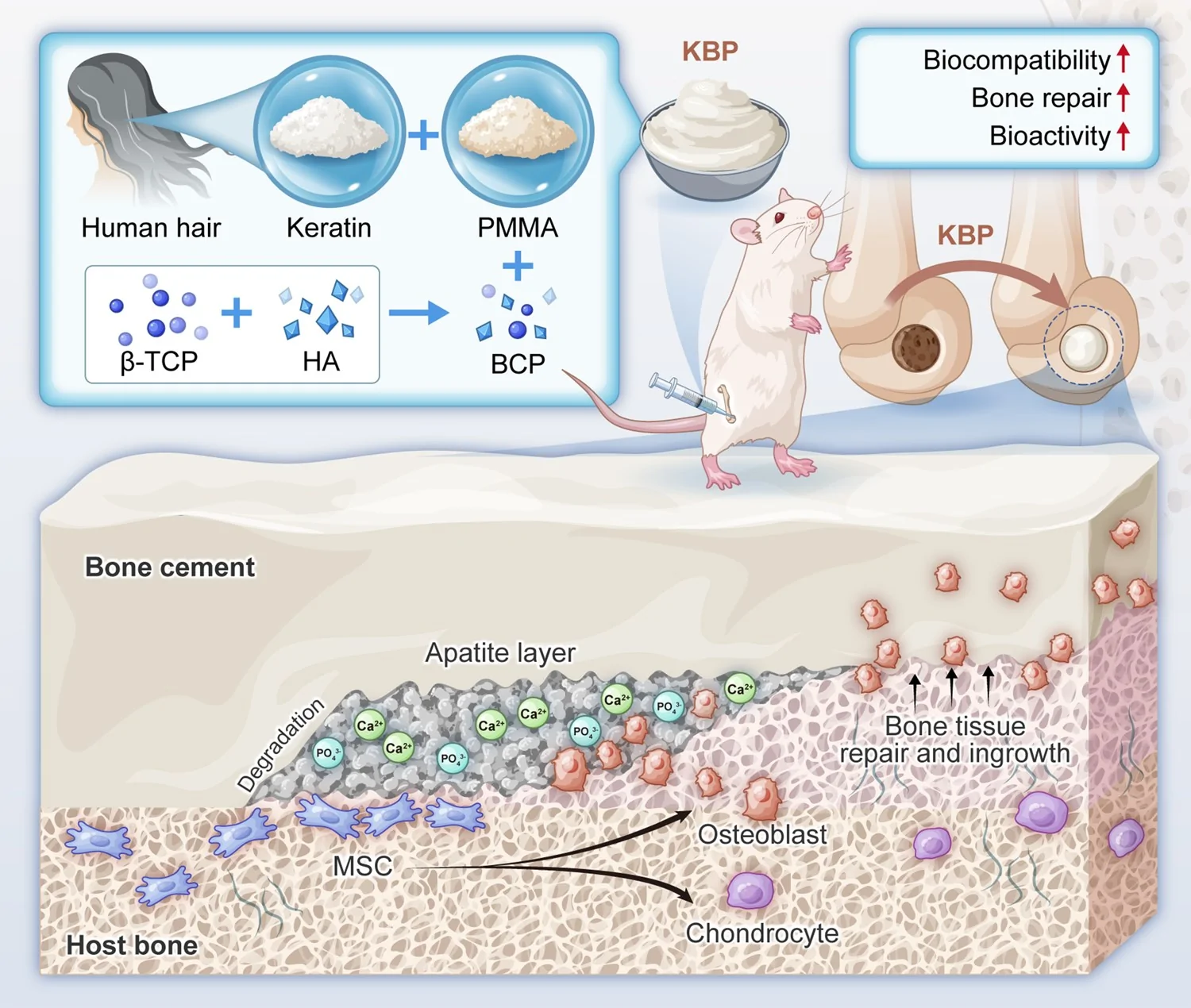
Schematic illustration for the underlying mechanism of human hair KBP bone cement in enhanced bone defect repair.

In this work, we present, for the first time, a human hair keratin-modified BCP/PMMA (KBP) bone cement with excellent biomedical properties (Figure 1). We investigated the effects of human hair keratin on the setting, mechanical, physical and biological properties of the cement. The proliferation and differentiation of MSCs were assessed *in vitro* through cell culture experiments using KBP, while *in vivo* biosafety and bone repair efficacy were evaluated in a Sprague–Dawley (SD) rat bone defect model. Results indicate that KBP bone cement exhibits improved handling properties, greater hydrophilicity, adequate mechanical strength and excellent biocompatibility. Moreover, it significantly facilitated the adhesion, proliferation and osteogenic and chondrogenic differentiation of human umbilical cord mesenchymal stem cells (HUMSCs), with *in vivo* tests showing that KBP markedly enhanced bone repair, presenting it as a viable alternative material for bone tissue repair.

## Results and discussion

### Physical and mechanical properties of BCP/PMMA bone cements

As shown in Figure 1, the BCP/PMMA was prepared by compounding solids of PMMA, HA and β-TCP with liquid MMA monomer at various compositions. Benzoyl peroxide (BPO) was used as an initiator to polymerize MMA with small amounts of auxiliary components such as ZrO_2_, N,N-dimethyl-p-toluidine (DMPT). The resulting BCP/PMMA bone cements were designated as BxPy, where “x” and “y” denote the mass ratios of BCP to solid PMMA within the bone cement composites, as specified in Table 1.

**Table 1:** Components of BCP/PMMA cement composites.

Bone cements	Solid component (g)				Liquid component (mL)	
	PMMA	BCP	BPO	ZrO_2_	MMA	DMPT
B0P10	10	0	0.125	0.8	4.95	0.05
B1P9	9	1	0.125	0.8	4.95	0.05
B2P8	8	2	0.125	0.8	4.95	0.05
B3P7	7	3	0.125	0.8	4.95	0.05
B4P6	6	4	0.125	0.8	4.95	0.05
B5P5	5	5	0.125	0.8	4.95	0.05

The average particle sizes for PMMA and BCP used in the preparation of bone cements were 82.6 and 7.4 µm, respectively, as shown in Supplementary Figures S1 and S2. The particle size plays a significant role in determining the mechanical properties and setting behavior of bone cements. Smaller particle sizes have been associated with enhanced mechanical properties, including improved compressive strength and more consistent setting times [26].

**Figure 2:**
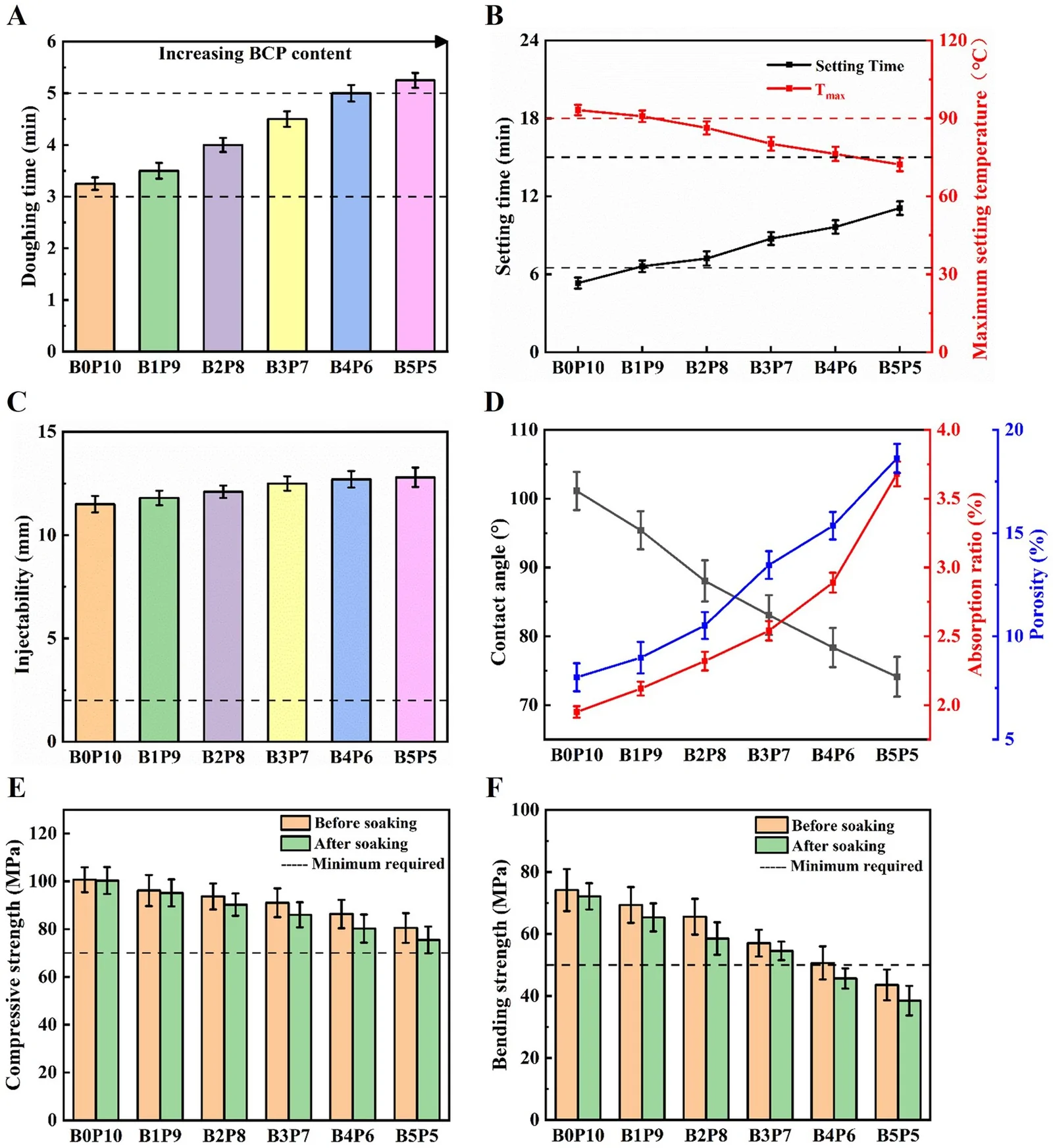
Physical and mechanical characterization of BCP/PMMA bone cements: (**A**) doughing time; (**B**) setting time and maximum setting temperature (*T*_max_); (**C**) injectability; (**D**) contact angle, absorption ratio and porosity; (**E**) compressive strength; (**F**) bending strength before and after soaking in SBF at 37°C for 28 days. The dashed lines represent the requirements specified in the international standard ISO 5833:2002.

The optimal timing for applying bone cement during surgery is identified as *just* before reaching the dough state, thus making doughing time a key parameter of proper handling and manipulation of bone cement in surgical use [27]. As illustrated in Figure 2A, the doughing time of BCP/PMMA bone cements increased as the BCP fraction increased. The doughing time of cement without BCP addition was 3.25 min, which extended to 5.25 min for the B5P5 composition (Figure 2A and Table 1). According to the International Standard ISO 5833:2002, the recommended doughing time for clinical use falls between 3 and 5 min, making B0P10, B1P9, B2P8 and B3P7 suitable formulations in terms of doughing characteristics.

The setting of bone cements describes their transition from fluid to rigid state, which is a critical process that directly influences their practical usability during surgery. The setting time and maximum setting temperature (*T*_max_) are two important factors in defining setting properties. Consistent with previous findings [28, 29], the setting time of BCP/PMMA bone cements tended to increase with higher BCP content (B3P7 8.8 min, B4P6 9.6 min and B5P5 11.1 min). This may be attributed to the retardation of the MMA polymerization rate after BCP incorporation, probably due to reduced reactivity and altered heat distribution within the cement matrix. The exothermic curing behavior is particularly important for PMMA-based bone cements because excessive heat release may injure surrounding tissue. Notably, BCP incorporation also attenuated the exothermic curing behavior of the bone cements, as reflected by the gradual decrease in *T*_max_ with increasing BCP content. Among the formulations meeting the ISO 5833:2002 requirements, *T*_max_ decreased from 80.2°C for B3P7 to 76.3°C for B4P6 and further to 72.2°C for B5P5 (Figure 2B), suggesting that BCP addition was beneficial for reducing heat accumulation during cement polymerization. According to ISO 5833:2002, the setting time should range from 6.5 to 15 min and *T*_max_ should not exceed 90°C, which was met by B3P7, B4P6 and B5P5, as shown in Figure 2B.

The injectability is another important factor, as it determines whether the cement can pass through narrow delivery channels smoothly, especially for minimally invasive procedures. Injectability was evaluated by measuring the penetration distance of bone cements from a perforated mold based on the extent of intrusion through a perforation, with a higher value indicating better injectability. As illustrated in Figure 2C, the increase in BCP content led to a slight improvement in injectability of bone cements (from 11.5 mm without BCP to 12.8 mm with 50% BCP), likely due to the associated decrease in viscosity as setting time increased. The spherical morphology of BCP and relatively small particle size might contribute to better dispersion within the PMMA matrix, further facilitating smoother injection [30, 31]. As previously discussed, the addition of BCP particles slowed the polymerization of MMA monomers, resulting in a lower viscosity and ultimately enhancing cement injection [32]. It is notable that all bone cements in this study sufficiently exceeded the minimum injectability of 2 mm specified in ISO 5833:2002. The doughing time, setting properties and injectability of the BCP/PMMA composites are comparable to those of previously reported PMMA-based cements [13], confirming that incorporation of BCP does not compromise the functional properties of bone cements but rather improves their usability for practical surgical settings.

Hydrophilicity and surface wettability, quantitatively reflected by the contact angle, can affect cell adhesion and proliferation [33]. Studies have found that bone cements with higher contact angles (relatively more hydrophobic surfaces) tend to hinder cell adhesion, which can adversely affect bone regeneration. In comparison, materials with lower contact angles (indicating higher hydrophilicity) are more conducive to cellular attachment and proliferation, as well as to the establishment of a stable interface between the implant and surrounding bone tissues [34, 35]. Since BCP particles are inherently hydrophilic and biocompatible [36], their addition can ameliorate the hydrophobicity of PMMA substrates. The contact angle of B0P10 was 110° and decreased to 74° in B5P5 (Figure 2D), demonstrating a clear shift toward more hydrophilic behavior after the inclusion of BCP particles. Improved hydrophilicity of biomaterial has been linked to better protein adsorption on cell surfaces, resulting in the subsequent attachment and growth of osteoblasts [37]. The presence of BCP moderately increased porosity, improving their specific surface area and thereby increasing the absorption ratio of simulated body fluid (SBF, Figure 2D). The roughness of the cement surfaces of the BCP was also increased (Supplementary Figure S3), a property known to promote osteointegration by providing more anchorage points for cell adhesion [38]. Taken together, the increased hydrophilicity, higher porosity and rougher micromorphology create a more favorable interface for bone tissue attachment and ingrowth.

Since implanted bone cement must support loads while promoting bone regeneration, matching mechanical properties with those of surrounding bones is important to avoid secondary injury [39]. Bone cements with mechanical profiles close to natural bone can distribute mechanical stress and would not cause implant-associated complications such as stress shielding and subsequent bone resorption [40, 41]. BCP/PMMA cements demonstrated reduced compressive strength and modulus as BCP content increased (Figure 2E and Supplementary Figure S4A). This may be caused by the decrease in specific surface area, as indicated by the increase in porosity (Figure 2D). The ISO 5833:2002 specifies that bone cements should possess a minimum compressive strength of 70 MPa. Both the PMMA cement (B0P10) and BCP/PMMA cement (B5P5) exceeded the requirement (100.7 and 80.52 MPa, respectively, orange bars in Figure 2E). Although pure PMMA offers higher strength, its rigidity may transfer excessive stress to adjacent bone, having the risk that leads to microfractures or implant failure, a phenomenon known as the stress shielding effect. Optimized composites like BCP/PMMA may overcome this challenge due to a balance of strength and flexibility [41]. The bending strength decreased with increased BCP addition (orange bars in Figure 2F), while the bending modulus increased (Supplementary Figure S4B). Exposure to SBF reduced mechanical performance in all formulations (green bars in Figure 2E and F), a trend consistent with previous studies showing that prolonged fluid exposure can compromise cement integrity [42]. Even so, B0P10, B1P9, B2P8 and B3P7 all met the ISO requirement of >50 MPa bending strength, both before and after soaking.

### Physical and mechanical properties of KBP

**Figure 3:**
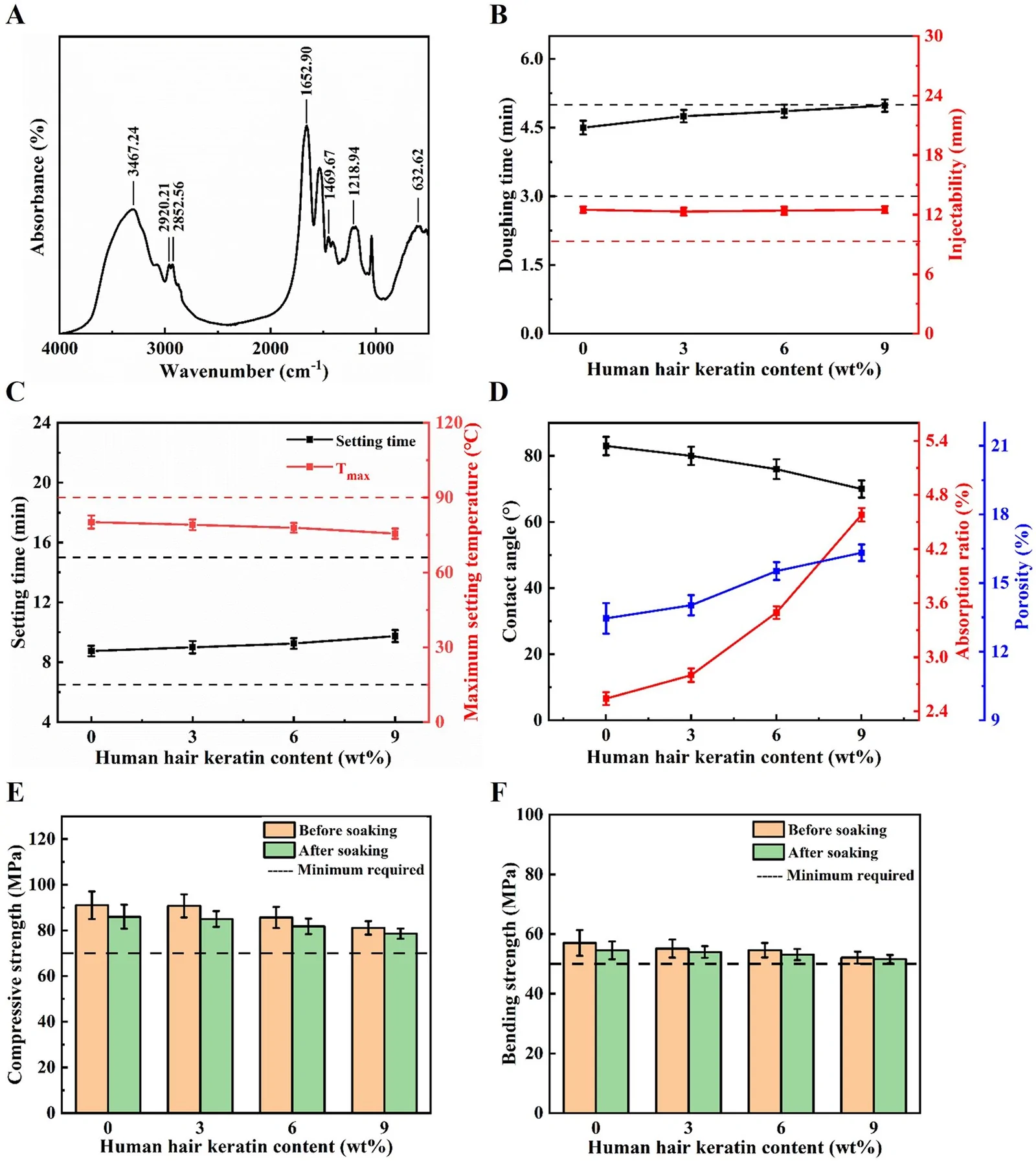
Physical and mechanical characterization of KBPs. (**A**) FTIR spectrum of human hair keratin; (**B**) doughing time and injectability; (**C**) setting time and maximum setting temperature (*T*_max_); (**D**) contact angle, absorption ratio and porosity; (**E**) compressive strength; and (**F**) bending strength of KBPs. The dashed lines represent the requirements specified in the international standard ISO 5833:2002.

With careful consideration of doughing time, setting properties, injectability, mechanical properties and *in vitro* biological properties, the B3P7 bone cement was selected for further studies. Extensive research has demonstrated the enhanced regeneration of the nervous system, tissues and somatic cells by keratin as a biomaterial for implantation [43]. The introduction of human hair keratin aimed to improve the biocompatibility and osteoconductivity of BCP/PMMA bone cements. The human hair keratin agglomerates used in this study had an average size of 25 μm, with morphologies depicted in Supplementary Figure S5. This particle size offers a practical balance between surface area and dispersibility within the PMMA matrix, helping to optimize both the mechanical performance and biological responsiveness of the composite. Figure 3A shows the Fourier transform infrared (FTIR) spectrum of human hair keratin, highlighting characteristic peaks mainly attributed to the vibration of peptide bonds (-CONH-) responsible for the bands of amides A, I, II and III [44]. Amide A, centered at 3467.24 cm^−1^, was associated with the stretching vibrations of N-H bonds. The strong Amide I band, assigned to C = O bonds, appeared at 1652.90 cm^−1^. The peak at 1469.67 cm^−1^ is related to the bending vibrations of N-H and the stretching vibrations of C-H bonds (Amide II band). Amide III, at 1218.94 cm^−1^, is mainly associated with the stretching vibrations of C-N and the bending vibrations of N-H and is partly related to the stretching vibrations of C-C and the bending vibrations of C = O. The out-of-plane bending vibrations of C-H bonds and the stretching vibrations of aldehyde groups resulted in characteristic peaks at 2920.21 and 2852.56 cm^−1^, respectively [45]. The adsorptions around 632.62 cm^−1^ are attributed to C-S stretching vibrations, confirming the presence of thiol groups found in keratins [46].

The incorporation of human hair keratin slightly extended the doughing and setting times of the B3P7 bone cement (Figure 3B and C). The inclusion of 9 wt% human hair keratin in B3P7 resulted in maximum doughing and setting times of 4.98 and 9.75 min, respectively. This extension can be attributed to the introduction of keratin particles, which may alter the polymerization kinetics and slightly slow the curing reaction. Meanwhile, *T*_max_ progressively decreased from 80.2°C for B3P7 to 75.6°C for B3P7-9 (Figure 3C), indicating that keratin incorporation further attenuated the exothermic curing behavior of BCP/PMMA bone cement. The injectability of B3P7 was weakly influenced by adding human hair keratin, fluctuating around 12.5 mm (Figure 3B). This may be ascribed to the balance between the increased viscosity caused by keratin particles and reduced polymerization rate, thereby retaining injectability. Overall, the doughing time, setting time and *T*_max_ of all KBP bone cements met the requirements of ISO 5833:2002.

The contact angle further decreased as more human hair keratin was incorporated, reaching 70° for KBP containing 9 wt% human hair keratin (Figure 3D). This improvement in hydrophilicity and wettability reflects the hydrophilic nature of both BCP and keratin. The addition of human hair keratin also increased the porosity of B3P7, from 13.46% to 16.32%. With higher porosity and a more hydrophilic surface, the SBF absorption ratio rose from 2.54% to 4.58%, thus promoting cell adhesion and growth. Together, these enhancements indicate that human hair keratin incorporation improves the operability of bone cements.

The compressive and bending strengths of KBP cements, either dry or soaked in SBF, decreased slightly with the increasing human hair keratin content (Figure 3E and F). With 9 wt% human hair keratin, the compressive strength fell from 91.04 MPa and 86 MPa for B3P7 to 81.1 MPa and 78.6 MPa for B3P7-9, before and after soaking, respectively. Bending strength decreased from 57.04 MPa and 54.53 MPa for B3P7 to 52.1 MPa and 51.6 MPa for B3P7-9, respectively. Although greater porosity can weaken the structure, it also improves biological performance—an effect reported in studies on biomaterial composites like keratin, where minor decreases in mechanical strength are often offset by significant gains in osteoconductivity and cytocompatibility [47, 48]. Despite these slight reductions, the mechanical properties of KBP bone cements remained above the minimum requirements. It has been suggested that maintaining mechanical properties within the acceptable range ensures that bone cements can still provide adequate load-bearing support during the bone healing process, consistent with recent work on composite biomaterials for orthopedic implants [49].

### Biological properties of KBP

#### *In vitro* biological properties of KBP

##### Biodegradation

**Figure 4:**
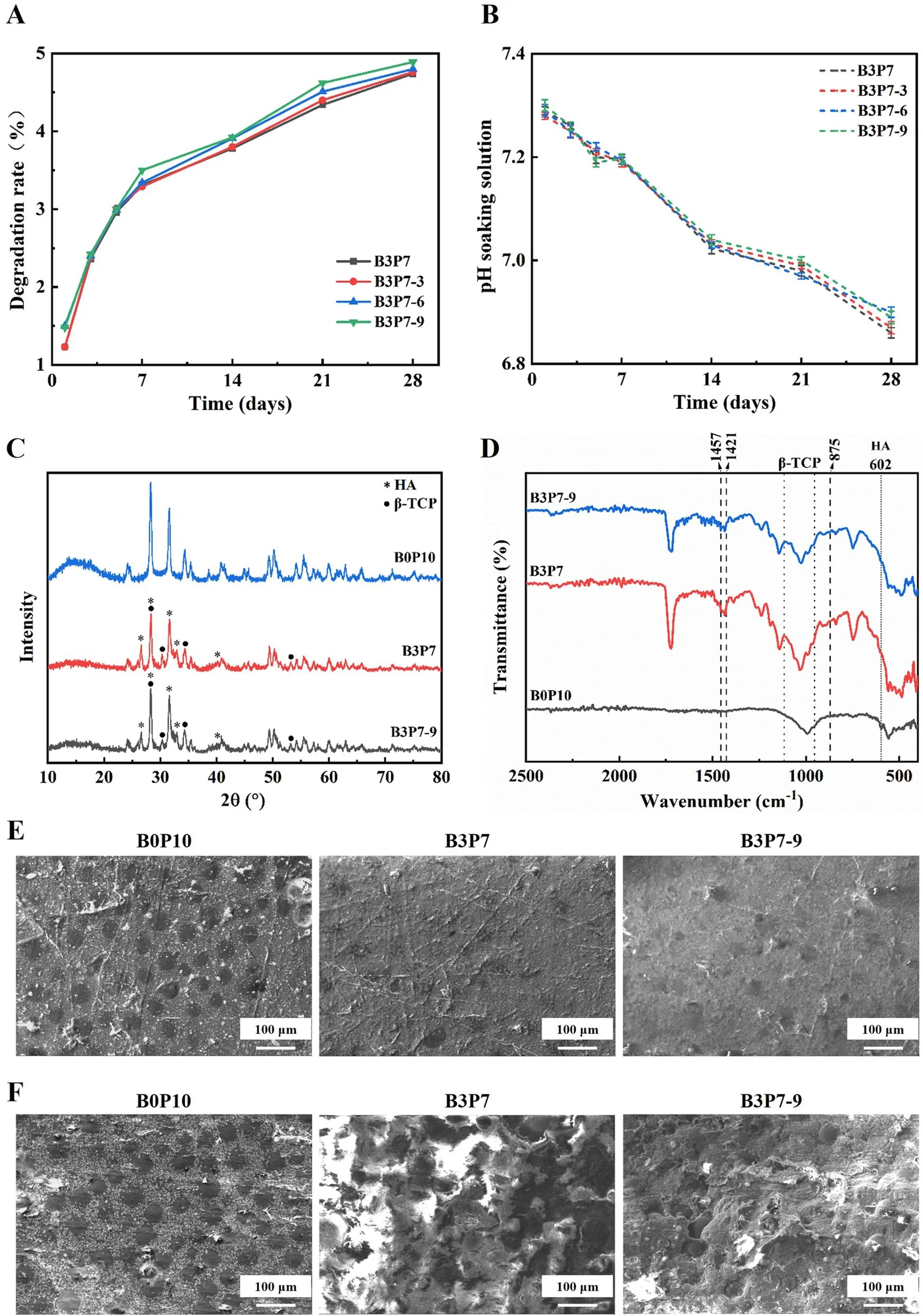
(**A**) Biodegradation rate of composite bone cements immersed in SBF for different durations; (**B**) pH value of the SBF soaking solution; (**C**) XRD and (**D**) FTIR spectra of different cements; surface morphologies of B0P10, B3P7 and B3P7-9 (**E**) before and (**F**) after degradation in SBF at 37°C for 28 days.

For biomaterials to form a stable bond with living bone, the development of a surface apatite layer is an important indicator of *in vitro* bioactivity and potential bone-bonding ability [50]. The apatite/HA layer creates a bioactive interface that resembles natural bone, supporting osteoblast attachment and proliferation and contributing to long-term implant stability. To evaluate the *in vitro* bioactivity of the composite bone cements, the samples were immersed in SBF for 28 days. As shown in Figure 4A, B3P7 and the human hair keratin-modified B3P7 samples exhibited comparable mass loss during immersion, indicating that keratin incorporation did not substantially alter the overall degradation behavior of the BCP/PMMA cement matrix. During SBF immersion, BCP dissolution and ion exchange can release calcium and phosphate ions and alter the pH of the soaking solution. The pH of the soaking solution gradually decreased during immersion (Figure 4B). Such a phenomenon of drop in pH is commonly observed in calcium phosphate-based cements, where BCP dissolution occurs concurrently with the release of calcium and phosphate ions [51].

To evaluate ion release, the concentrations of Ca^2+^ and PO_4_³^−^ in the SBF soaking solutions were measured by inductively coupled plasma optical emission spectroscopy (ICP-OES, Supplementary Figure S7). B0P10 showed only minimal Ca^2+^ and PO_4_³^−^ release throughout the 28-day immersion period. In contrast, the BCP-containing B3P7 and B3P7-9 groups exhibited markedly higher ion concentrations. On Day 28, the Ca^2+^ concentrations of B3P7 and B3P7-9 reached 0.699 ± 0.011 and 0.737 ± 0.027 mM, respectively, while the PO_4_³^−^ concentrations reached 0.286 ± 0.013 and 0.361 ± 0.033 mM, respectively. These results confirm that BCP degradation contributes to the release of calcium and phosphate ions, providing an ion-rich microenvironment that supports apatite formation and the bioactivity of BCP/PMMA and KBP bone cements. To further clarify the degradation contribution of keratin, the soluble keratin-derived protein/peptide products released from B3P7, B3P7-3, B3P7-6 and B3P7-9 were quantified during SBF immersion (Supplementary Figure S8). B3P7 without keratin showed only a low background level throughout the immersion period. In contrast, the keratin-containing groups exhibited a gradual increase in soluble degradation product concentration over time. The released product concentration also increased with increasing keratin content, with B3P7-9 showing the highest level after 28 days of immersion. These results indicate that keratin in the KBP cements undergoes gradual degradation in SBF and releases soluble protein/peptide products, which may contribute to the bioactive microenvironment of the cement.

The apatite-forming ability of the cements after SBF immersion was further examined by X-ray diffraction (XRD) and FTIR spectroscopy. As shown in Figure 4C, B3P7 and B3P7-9 exhibited diffraction peaks corresponding to calcium phosphate phases after 28 days of soaking in SBF, while the B0P10 group did not. The peaks at 26°, 28°, 32°, 33° and 40° were assigned to HA, and the peaks at 28°, 30°, 34° and 53° were characteristic of β-TCP. The intensity of HA and β-TCP in the B3P7-9 group was slightly higher than that in the B3P7 group. In the FTIR spectra (Figure 4D), new bands at 1410 and 1452 cm^−1^, assigned to CO_3_^2−^, were observed on the surface of soaked BCP/PMMA bone cements, suggesting the formation of carbonated HA or bone-like apatite. According to the literature, the formation of carbonated HA or bone-like apatite on the surface of implanted materials during degradation is an important criterion for evaluating their biological activity [52]. These results demonstrate that BCP incorporation endows PMMA bone cement with osteoconductive potential. The surface morphologies of bone cements before and after SBF immersion were characterized by scanning electron microscopy (SEM, Figure 4E and F). As expected, little change was observed on the surface of B0P10 cement after immersion in SBF due to the bioinert nature of PMMA. In contrast, the BCP-containing cements displayed obvious surface erosion and mineral-like deposits after 28 days of immersion, which further supported BCP dissolution and apatite-related mineralization. Together with the mass loss, pH change, Ca^2+^/PO_4_³^−^ release, soluble keratin-derived degradation product release and apatite-related characterization results, these findings confirm that BCP and keratin contribute to the degradation behavior of KBP through complementary pathways, thereby improving the bioactivity of PMMA-based bone cement.

##### Cell viability

**Figure 5:**
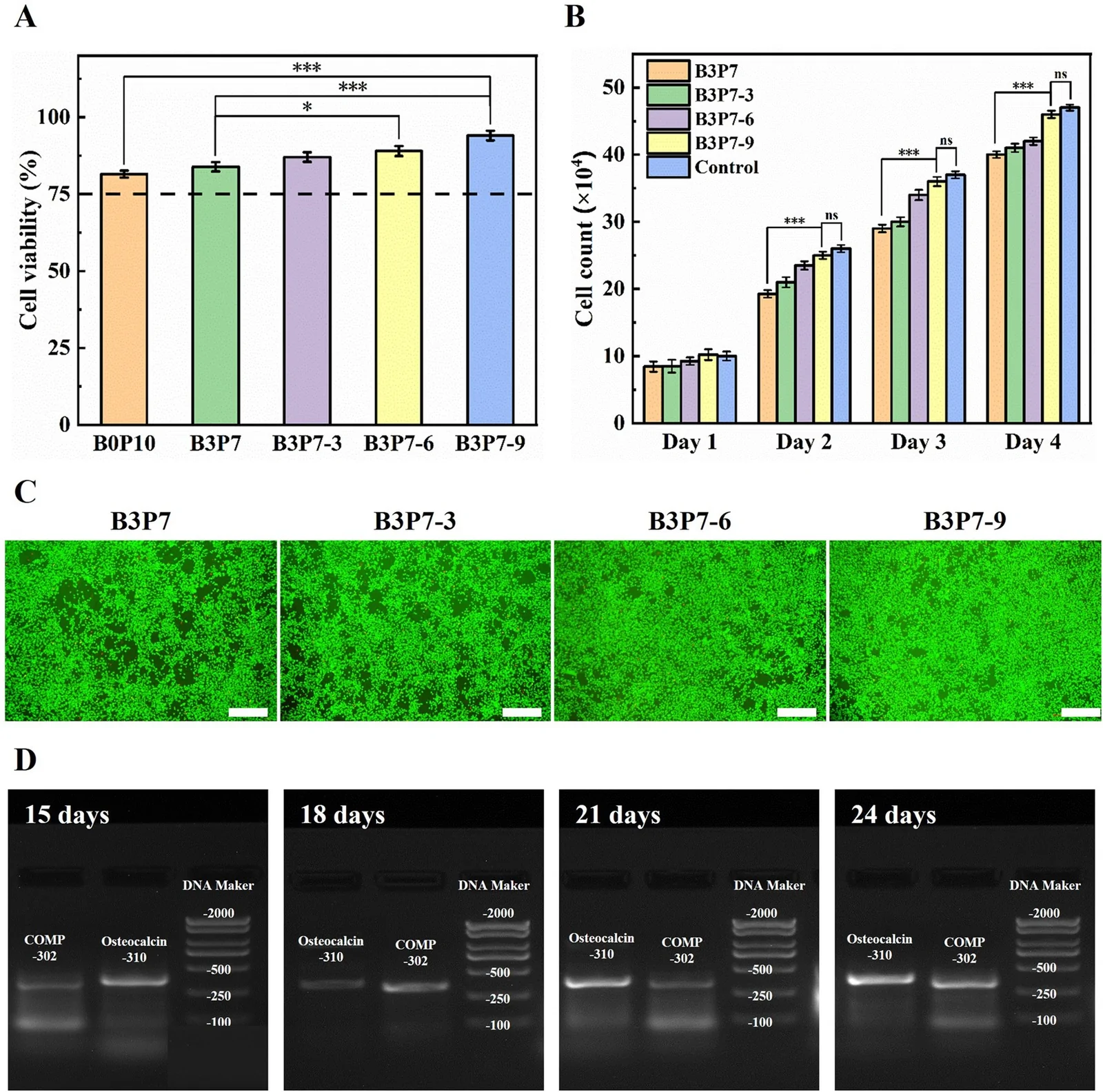
(**A**) Cell viability after 5 days of HUMSCs cultured in different bone cement extracts; (**B**) HUMSC numbers during 4 days of *in vitro* coculture; (**C**) live/dead cell staining images, scale bar: 500 µm; (**D**) expression of osteocalcin and COMP in HUMSCs on Days 15, 18, 21 and 24 of coculture with the B3P7-9 bone cement. *P < 0.05, **P < 0.01, ***P < 0.001.

Artificial bone materials should be highly biocompatible to support cell adhesion, proliferation, differentiation and bone regeneration. HUMSCs were used to evaluate the cytocompatibility of KBP bone cements. Based on their setting behavior, mechanical properties and possible biocompatibility, B3P7, B3P7-3, B3P7-6 and B3P7-9 were selected as representative formulations for further evaluation. The cytotoxicity of the cement extracts was assessed by the methyl thiazolyl tetrazolium (MTT) assay. B0P10 showed a cell viability of 81.5%, whereas B3P7 exhibited increased cell viability, indicating that the incorporation of BCP improved the cytocompatibility of PMMA cement (Figure 5A). With the further incorporation of human hair keratin particles, cell viability increased gradually, and B3P7-9 exhibited the highest viability among all cement groups, reaching 94%. According to the cytotoxicity classification shown in Supplementary Table S1, all KBP formulations were classified as level 1 cytotoxicity.

Cell proliferation was further evaluated by counting HUMSCs cultured with different cement extracts from Day 1 to Day 4. As shown in Figure 5B, cell numbers increased continuously over time in all groups, indicating that the cement extracts did not inhibit HUMSC proliferation. Among the cement groups, B3P7-9 consistently showed the highest cell counts. From Day 2 to Day 4, B3P7-9 resulted in significantly higher cell numbers than B3P7 and showed no significant difference compared with the control group, suggesting that the incorporation of human hair keratin improved the proliferation-supporting capacity of the cement. These results demonstrate that the combined incorporation of BCP and human hair keratin improves the cytocompatibility of PMMA-based bone cement and supports HUMSC proliferation.

A live/dead staining assay was performed after culturing HUMSCs with sterilized bone cements for 4 days (Figure 5C). In the merged images, abundant green fluorescence and few red-stained dead cells were observed in all groups, indicating good cell survival after coculture with the bone cements. Compared with B3P7, the keratin-containing groups showed denser live-cell staining, with B3P7-9 displaying the most favorable cell distribution, which was consistent with the MTT and cell-count results. These findings further confirm that human hair keratin modification enhances the cytocompatibility of BCP/PMMA bone cement by supporting HUMSC survival and proliferation.

##### Expression of osteogenic and chondrogenic markers

To assess the potential of KBP bone cements to induce osteogenic and chondrogenic differentiation of HUMSCs, RNA was extracted from cells co-cultured with B3P7-9 for 15, 18, 21 and 24 days. Reverse transcription polymerase chain reaction (RT-PCR) was then performed to amplify genes for osteocalcin and cartilage oligomeric matrix protein (COMP), which are expressed by osteocytes and chondrocytes, respectively. Figure 5D shows that both osteogenic and chondrogenic markers were detected on Day 15, and the intensities of both bands increased as the co-culture time was extended to 24 days, indicating elevated expression levels.

To further verify the osteogenic and chondrogenic differentiation potential of B3P7-9, Alizarin red S and Toluidine blue staining were performed after 21 days of culture (Supplementary Figure S6). Compared with the control group, the B3P7-9 group showed more evident Alizarin red S staining, indicating enhanced calcium deposition. In addition, stronger toluidine blue staining was observed in the B3P7-9 group, suggesting increased formation of an acidic mucopolysaccharide-rich matrix. These staining results were consistent with the RT-PCR results and further confirmed that B3P7-9 promoted osteogenic and chondrogenic differentiation of HUMSCs.

The ability of B3P7-9 bone cements to induce osteogenic and chondrogenic differentiation may be attributed to the combined effects of BCP and human hair keratin. First, the degradation of BCP in B3P7-9 composites promoted the formation of bone-like HA, which is beneficial for protein adsorption and cell adhesion. In addition, BCP degradation can provide calcium and phosphate ions that support osteoblast proliferation, maturation and osteogenic differentiation. Second, human hair keratin provides abundant cell-interactive motifs and an extracellular matrix-like microenvironment, which may facilitate HUMSC adhesion, survival and lineage-specific differentiation. Previous studies have reported that human hair keratin can promote osteogenesis-related gene expression in stem cells [24, 25], while keratin-based scaffolds can also support chondrogenic differentiation and cartilage-like matrix formation [22]. Therefore, the enhanced osteocalcin expression, COMP expression, Alizarin red S staining and Toluidine blue staining observed in the B3P7-9 group may result from the synergistic contribution of BCP-derived mineral bioactivity and keratin-mediated biological cues.

#### *In vivo* biological properties of KBP

##### Micro-CT evaluation

**Figure 6:**
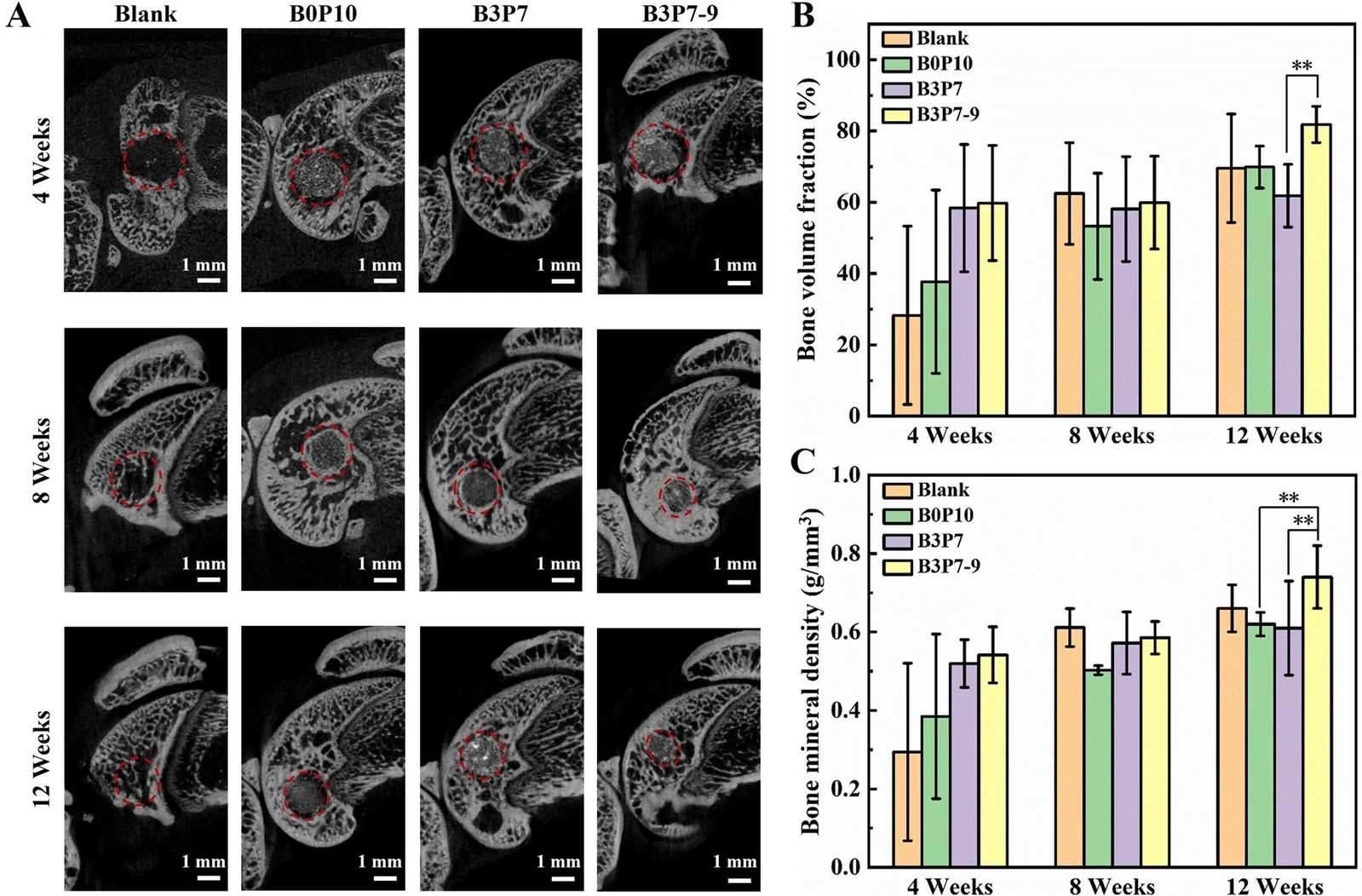
(**A**) Micro-CT images showing the progression of bone healing over time at 4, 8 and 12 weeks postsurgery. Red dashed circles indicate the location of the bone defects. Quantitative analysis of (**B**) bone volume fraction (BV/TV) and (**C**) BMD in different treatment groups at 4, 8 and 12 weeks postsurgery. *P < 0.05, **P < 0.01, ***P < 0.001.

An SD model of distal femur defects with a diameter of 2 mm and a depth of 2 mm was established to evaluate the bone-repairing ability of the cements. The formation of new bone tissue was assessed using microcomputed tomography (micro-CT) images for each group at 4, 8 and 12 weeks postsurgery (Figure 6). In the pure PMMA cement group, there was no significant change over time, and the interface between the cement and bone tissue remained distinct throughout the study period. In contrast, the BCP/PMMA and KBP groups showed partial absorption of the cement and new bone formation.

After 12 weeks postsurgery, the bone volume fraction (bone volume/total volume, BV/TV) values in the empty defect, PMMA, BCP/PMMA and KBP groups were 69.53%, 69.90%, 61.97% and 80.39%, respectively (Figure 6B). The local bone mineral density (BMD) values were 0.656 g/mm^3^, 0.621 g/mm^3^, 0.605 g/mm^3^ and 0.727 g/mm^3^, respectively (Figure 6C). Quantitative analysis showed that the KBP group exhibited a significantly higher BV/TV than the BCP/PMMA group at 12 weeks postsurgery (*P* < 0.01). In addition, the BMD value of the KBP group was significantly higher than those of the PMMA and BCP/PMMA groups (*P* < 0.01). These results indicate that the incorporation of keratin into BCP/PMMA bone cement promotes new bone formation.

##### Histological staining

**Figure 7:**
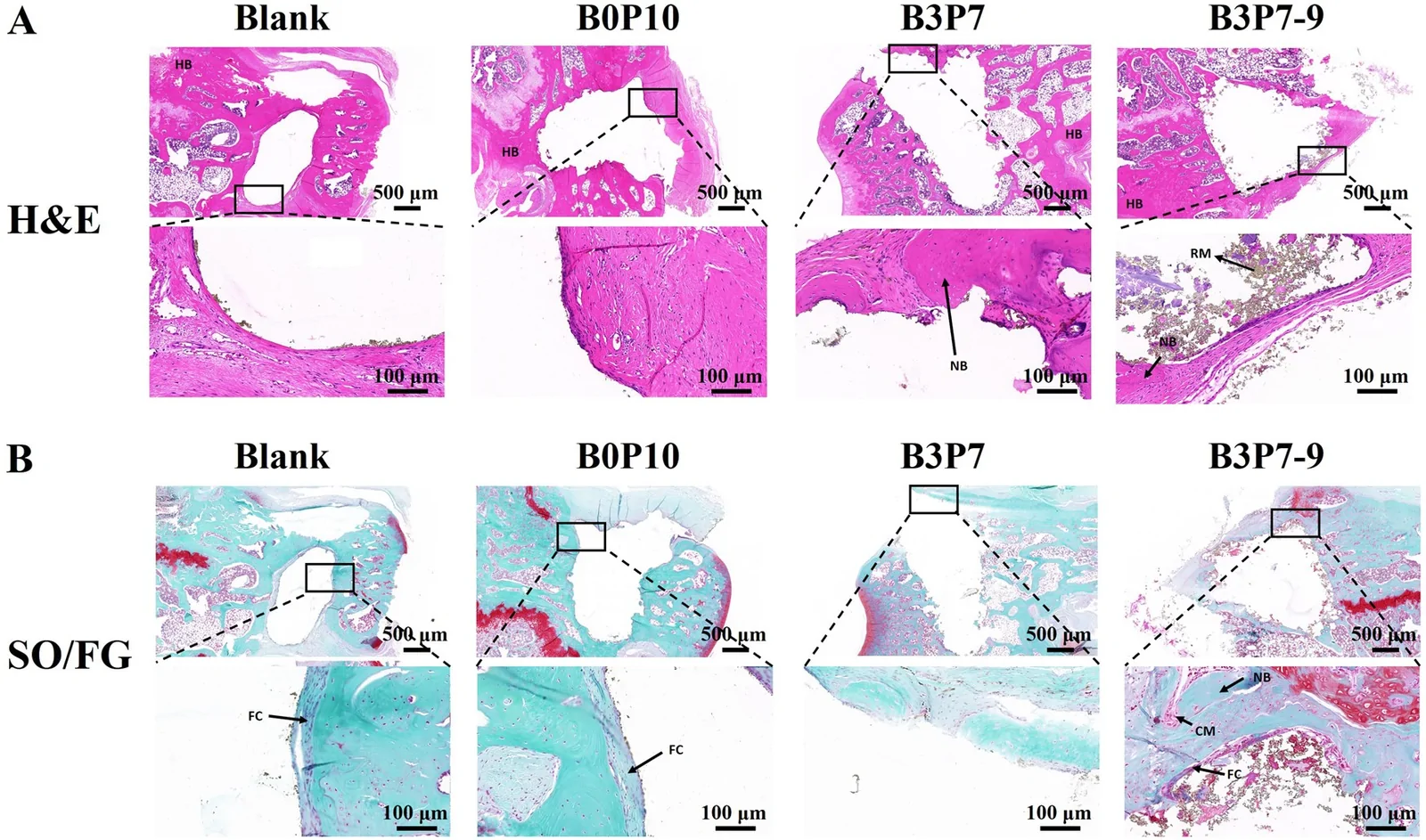
Histological evaluation of bone repair at 12 weeks by (**A**) H&E and (**B**) SO/FG staining. Representative annotated regions are indicated as follows: HB, host bone; FC, fibrous capsule; NB, newly formed bone; RM, residual material; and CM, cartilage-like matrix.

Representative hematoxylin and eosin (H&E) and Safranin O-fast green (SO/FG) staining images are presented in Figure 7. In the blank group, the defect area remained largely unfilled and was surrounded by dense fibrous connective tissue, suggesting limited spontaneous repair. In the B0P10 group, the relatively continuous bony margin was observed around the defect, with less fibrous encapsulation than in the blank group, although the central defect space remained evident. In the B3P7 group, fibrous encapsulation was less obvious, whereas callus formation and bone matrix deposition were discontinuous and immature. By contrast, the B3P7-9 group showed a more complete callus and newly formed bone layer along the defect margin, and SO/FG staining revealed cartilage-like matrix in the repair region. Only scattered inflammatory cells, mainly macrophages and multinucleated cells, were observed at the defect margins in all groups, and no severe inflammatory reaction was detected. These histological observations are consistent with the micro-CT results and further indicate that keratin incorporation improved the bone-repairing capacity and tissue compatibility of BCP/PMMA bone cement.

##### In vivo biocompatibility

**Figure 8:**
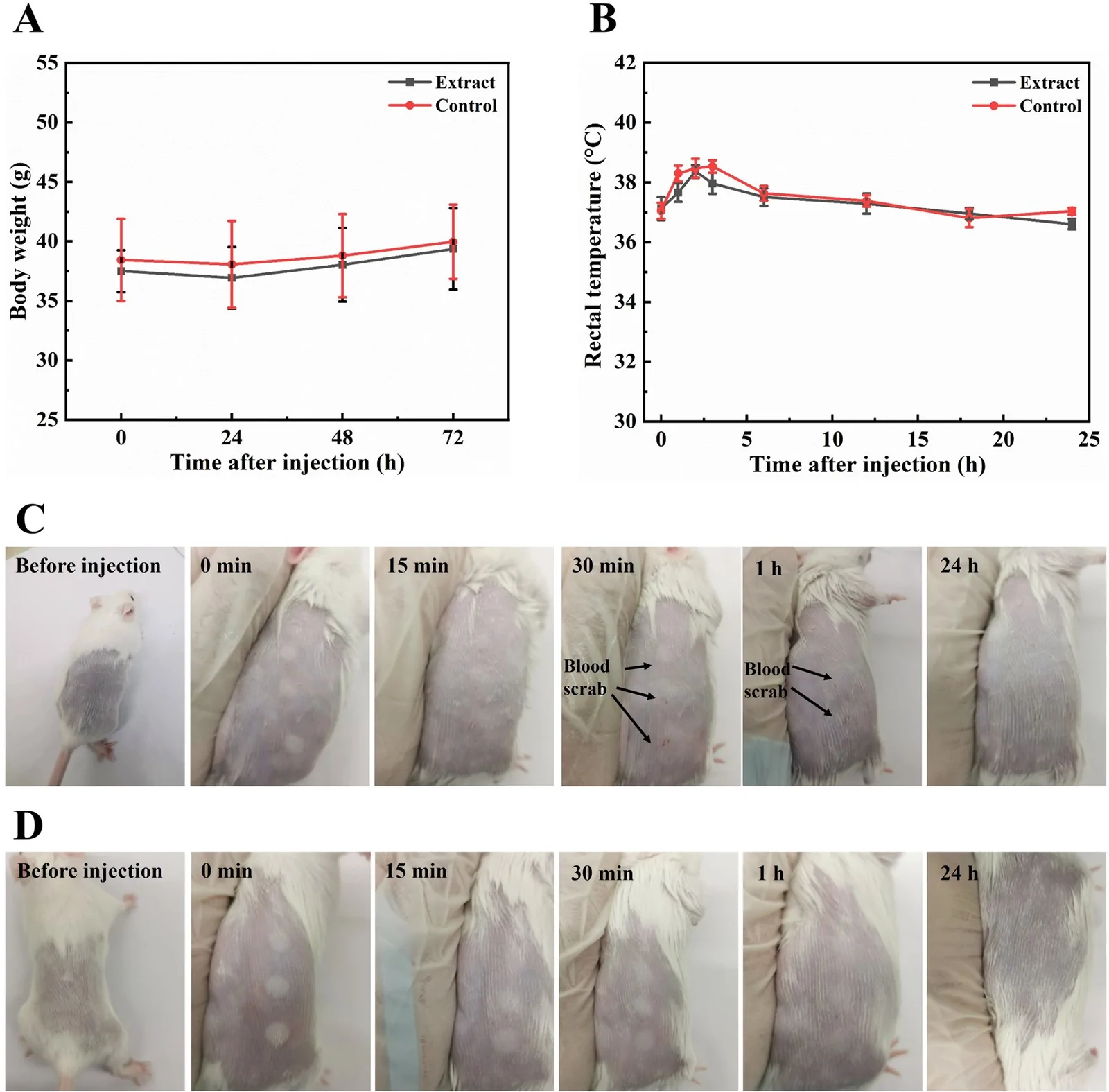
*In vivo* biocompatibility of KBP. (**A**) Body weight and (**B**) rectal temperature of rats before and after injection of B3P7-9 extracts. Sensitization tests on mice injected with (**C**) B3P7-9 extracts and (**D**) saline.

After evaluating the *in vivo* biological properties of KBP cements, B3P7-9 was selected for further *in vivo* biocompatibility assessment. The results of the acute systemic toxicity test are presented in Figure 8. All mice that received abdominal cavity injections of B3P7-9 cement extracts survived the duration of the experiment without exhibiting any adverse effects, such as febrile convulsions, hemiplegia or respiratory depression. Moreover, mice administered B3P7-9 extracts gained an average weight of 1.46 g (from 38.44 to 39.90 g), while the negative control group had an average weight gain of 1.87 g (from 37.50 to 39.37 g, Figure 8A). These differences were not statistically significant, indicating that the extracts of B3P7-9 bone cements were nontoxic *in vivo*.

The introduction of exogenous substances into the body has the potential to trigger pyrogenic reactions with symptoms including elevated body temperature, chills and nausea. A pyrogenicity test was conducted to further evaluate the biosecurity of KBP bone cements. After tail-vein injection of extracts from B3P7-9, all SD rats remained alive throughout the experiment without any symptoms of excitement, inattention, depression, reduced body activity, mania or inappetence. Shortly after administration, rectal temperature increased in both the test and control groups and then gradually returned to normal levels (Figure 8B). There was no significant difference in temperature profiles between the two groups, indicating that the B3P7-9 extract did not provoke a pyrogenic response *in vivo*. This result further demonstrated the biocompatibility of KBP cement, which is nonpyrogenic and unlikely to induce immunological responses that may endanger patient safety.

The *in vivo* sensitization potential of the B3P7-9 formulation was also assessed, as illustrated in Figure 8C and D. After 30 min and 1 h, small blood scabs formed at the injection sites in mice receiving the B3P7-9 extract, but these changes resolved within 24 h (Figure 8C). More importantly, there were no erythema, edema or induration observed (Figure 8D), suggesting that no significant inflammatory or allergic reactions have been induced. The mice behaved normally and showed little difference from saline-treated controls. According to the scoring criteria in Supplementary Table S3, the level of sensitization for B3P7-9 was therefore classified as level 0, meaning that the extract did not elicit a skin-sensitizing effect. Taken together, these findings show that KBP bone cements are nontoxic, nonpyrogenic and free of allergic sensitization risks, which further substantiates their promise for safe biomedical application.

Compared with other PMMA bioactivation strategies, including calcium phosphate-modified PMMA, graphene- or carbon nanotube-based PMMA composites and magnesium phosphate/HA-modified PMMA systems [4, 6–10, 13], the KBP formulation provides a balanced combination of mechanical reliability and biological activity. Calcium phosphate fillers have been widely used to improve the osteoconductivity and bioactivity of PMMA-based cements, but high inorganic incorporation may be accompanied by tradeoffs in mechanical performance or may only partially address the limited cytocompatibility of PMMA [8–10, 13]. Carbon-based or other inorganic nanoscale additives can enhance selected mechanical, thermal, antimicrobial or surface-related properties, but their performance is strongly dependent on additive composition, dispersion and interfacial compatibility within the cement matrix [4, 6, 7]. In this study, KBP retained compressive and bending strengths above the ISO 5833:2002 requirements while reducing the maximum setting temperature, improving hydrophilicity and porosity, supporting cell survival and promoting osteogenic/chondrogenic differentiation and *in vivo* bone repair. Therefore, the combined BCP/keratin design offers a complementary bioactivation strategy in which BCP provides ion-mediated mineral bioactivity, while human hair keratin supplies cell-interactive biological cues that improve cell-material interactions [16–18, 24, 25].

## Conclusion

In summary, this human hair KBP composite bone cement showed excellent biocompatibility, bioactivity and osteogenic properties, positioning it as a promising candidate for bone repair and regeneration. The incorporation of human hair keratin into the BCP/PMMA system not only improved doughing and setting properties (e.g. *T*_max_ deceased from 80.2°C to 75.6°C) but also markedly enhanced injectability and operability, which are important factors for practical use in surgery. In addition, the combination use of BCP and human hair keratin helped to counteract the poor fusion of PMMA with surrounding bone tissue while maintaining mechanical strength. Thus, KBP bone cements offer a unique balance of mechanical robustness and biological activity.

Improvements in hydrophilicity (contact angle decreased from 83° to 70°), porosity (from 13.5% to 16.3%) and SBF absorption (from 2.54% to 4.58%) collectively promoted cell adhesion and tissue ingrowth, which was reflected in a substantial increase in cell viability from 81.5% to 94%. The biological responsiveness was promoted after adding BCP and human hair keratin, as evidenced by the increase in expression of osteocalcin and COMP, which are markers of osteogenic and chondrogenic differentiation, respectively. During degradation, HA in bone cement releases calcium and phosphorus ions into the microenvironment, making it favorable for bone cell differentiation, proliferation and adhesion, while human hair keratin promotes bone and cartilage differentiation and enhances overall cell cytocompatibility.

*In vivo* studies confirmed that 9% human hair keratin promoted bone repair in an SD rat bone defect model, evidenced by the increase in both bone volume fraction from 69.9% to 80.4% and BMD from 0.62–0.73 g/mm^3^. Moreover, the KBP composites exhibited nontoxic, nonpyrogenic and nonsensitizing features. Overall, this work highlights the important role of human hair keratin in enhancing the biocompatibility, bioactivity and osteogenic properties of bone cements, offering a new approach in the design of advanced biomaterials for bone repair and regeneration.

## Experimental section

### Materials

PMMA bone cement powder, HA nanoparticles, β-TCP and methyl methacrylate (MMA) were purchased from Biochem Safebuy (Wuhan, China). DMPT, BPO and zirconium dioxide (ZrO_2_) were obtained from Beijing Honghu Lianhe Huagong Chanpin Co., Ltd (Beijing, China). SBF was purchased from Coolaber (Beijing, China). Human hair was collected from a local hair salon with appropriate permissions. HUMSCs were provided by The First Affiliated Hospital of Baotou Medical College, while SD rats were obtained from Beijing Vitonglihua Experimental Animal Technology Co., Ltd.

### Preparation of BCP/PMMA bone cements

HA and β-TCP powder were taken in the mass ratio of 3:7. The two powder ingredients were mixed using agate balls in a planetary ball mill. The mixture was ground at 500 rpm for 6 h and then screened through a 100-mesh filter to obtain BCP powder. Finally, the powder was sealed in a sterile bottle.

Bone cements composed of BCP and PMMA were prepared by mixing solid and liquid constituents (see detail in Table 1). The solid phase includes BCP, PMMA, BPO and ZrO_2_. BPO is the initiator of polymerization and ZrO_2_ is a radiopacifier. After mixing, polymerization occurs, and the cement undergoes the process of curing. Liquid components included MMA and DMPT, in which MMA is the monomer, and DMPT is an accelerator.

### Preparation of KBPs

The collected human hair was first washed with deionized (DI) water and de-lipidized in chloroform/methanol (2:1 v/v) solution. For oxidative bleaching, human hair (5 g) was sequentially mixed with sodium pyrophosphate (8 g), potassium persulfate (3 g), ammonia solution (25 mL, 25%) and hydrogen peroxide (75 mL, 30%) in a beaker. The reaction was conducted in a water bath at room temperature for 1.5 h. The treated hair was then thoroughly washed with DI water, dried at 50°C, and cut into 1-3 mm pieces. Subsequently, the hair fragments were ground in a Tencen Power XQM-2L planetary ball mill (Changsha, China) at 550 rpm, 10–15°C for 2 h to obtain human hair keratin powder. Different amounts of keratin powder were introduced into B3P7 to form keratin-modified B3P7 composites, which were denoted as B3P7-z, where “z” represents the weight percentage of human hair keratin.

### Particle size distribution

The particle size distribution of PMMA and BCP was measured using particle size analyzer (LS-609, OMEC). The surface wettability of bone cements was measured with goniometer (Hukuto CA60, Guangdong, China). Contact angles were measured by dropping water droplets onto samples after 3 s to be stable. The SBF absorption ratio (*A_r_*) was analyzed by immersing dry bone cements in SBF at 37°C for 2 min and was calculated by the equation:


(1) $$A_{r}\;(\%)=\frac{M_{n}-M_{o}}{M_{o}}\times 100\%,$$

where *M_o_* and *M_n_* are weights before and after immersing in SBF, respectively. Porosity (*P*) was investigated using the Archimedes drainage method in SBF solution and calculated using the equation:


(2) $$P=\frac{m_{2}-m_{1}}{m_{2}-m_{3}}\times 100\%,$$

where *m_1_* is the dried weight, *m_2_* is the saturated wet weight in air and *m_3_* is the saturated weight in liquid.

### Setting, mechanical and injectable properties

The setting, mechanical and injectable properties of the bone cements were tested according to the International Standard ISO 5833:2002. The setting properties of the bone cements with and without the addition of human hair keratin were evaluated by measuring the doughing time, setting time and maximum setting temperature (T_max_). All tests were performed at room temperature (23 ± 1°C) and a relative humidity of at least 40%. The doughing time is defined as the time from mixing solid and liquid phases until the finger touching the cement can be separated from the cement without cement sticking to it. The cement mixture surface was gently touched at 15 s intervals, and time was recorded starting immediately after mixing. The T_max_ was determined by inserting a nickel/chromium/aluminum thermocouple thermometer into the center of thoroughly mixed cement. The setting time was calculated as half of the time required to reach *T*_max_.

The compressive strength of bone cements was tested by compressing cylindrical specimens (6 mm in diameter, 12 mm in length) in the radial direction using a WDW-200 universal mechanical testing machine (Geling Technologies, Guangzhou, China), with a loading rate of 20 mm/min. For bending properties, rectangular specimens (75 mm × 10 mm × 3.3 mm) were tested using a three-point bending setup at a crosshead speed of 5 mm/min. The injectability of bone cements was quantitatively evaluated by measuring how much of the cement could be pushed through the perforation of a bottom-face-perforated mold when a 49 N force was applied for 1 min following doughing for 1 min.

### Biodegradation

The biodegradation of BCP/PMMA bone cements was evaluated by soaking in SBF (pH 7.4) at 37 ± 1°C. Periodically, the residual weights of the cements and the pH values of the SBF were measured to evaluate the degradation performance. After 28 days of immersion, the surface morphologies and mineral layers on the cement were examined using SEM (Quanta650, FEI), XRD (Bruker, D8 ADVANCE, German) and FTIR spectroscopy (Tensor II, Bruker, Beijing) to assess biomineralization.

Ion release analysis: The release of Ca^2+^ and PO_4_³^−^ from bone cements was evaluated in SBF. Briefly, B0P10, B3P7 and B3P7-9 samples were immersed in SBF at 37°C. At predetermined time points of 1, 3, 7, 14, 21 and 28 days, the soaking solutions were collected. The concentrations of Ca^2+^ and PO_4_³^−^ were measured by ICP-OES.

Keratin degradation product release analysis: B3P7, B3P7-3, B3P7-6 and B3P7-9 samples were immersed in sterile SBF at a solid/liquid ratio of 0.2 g/mL and incubated at 37°C. At predetermined time points of 1, 3, 7, 14, 21 and 28 days, the soaking solutions were collected and replaced with an equal volume of fresh SBF. The soluble keratin-derived degradation products in the collected solutions were quantified using a NanoDrop 2000 spectrophotometer in the Protein A280 mode, with fresh SBF used as the blank control.

### Cytotoxicity

The cytotoxicity of bone cement composites was evaluated using the MTT assay and live/dead assay. Sterilized bone cements were incubated at a concentration of 0.2 g/mL in Minimal Eagle Medium α (α-MEM, 8122210, Gibco) at 37°C for 72 h to obtain bone cement extracts, which were subsequently stored at 4°C. HUMSCs were cultured in bone cement extracts at 37°C in a humidified atmosphere containing 5% CO_2_. The cells were then seeded into a 96-well plate, with bone cement extracts (100 μL, 5 × 10^3^ cells/mL) in each well. Concurrently, control wells (containing both medium and cells) and blank wells (containing only medium) were established in each group. MTT assays were performed on Day 5 of culture, following the instructions provided by the manufacturer (Solarbio). Absorbance (Abs) was measured at 490 nm using a microplate reader (MulitiskanGo1510, Thermo). The cell viability was calculated as follows:


(3) $$\mathrm{Cell}\;\mathrm{viability}=\frac{{\mathrm{Abs}}_{\mathrm{extract}}-{\mathrm{Abs}}_{\mathrm{blank}}}{{\mathrm{Abs}}_{\mathrm{control}}-{\mathrm{Abs}}_{\mathrm{blank}}}\times 100\%,$$

where Abs_blank_ is the Abs of α-MEM, while Abs_extract_ and Abs_control_ refer to the Abs of the culturing solution with and without bone cement extracts, respectively. The cytotoxicity was determined according to the cell viability as specified in Supplementary Table S1.

For the live/dead assay, sterilized bone cement samples (0.5 cm^3^) were cocultured with HUMSC cell suspension (2 mL, 2 × 10^4^ cells/well) in a 24-well plate. After 4 days of incubation, the cells were stained using a live/dead cell double staining kit (KTA001, Abbkine) and examined with an inverted fluorescence microscope (Ti-S, Nikon). Cell proliferation over the 4-day culture period was quantified using a phase contrast microscope (XDS-1A, Cany Precision Instruments Co., Ltd, Shanghai, China).

### Gene expression and staining analysis

For gene expression analysis by RT-PCR, sterilized bone cements (0.5 cm^3^) were cocultured with HUMSC cell suspension (2 mL, 2 × 10^4^ cells/well) in a 24-well plate using α-MEM supplemented with 10% fetal bovine serum (FBS). After 15, 18, 21 and 24 days of culture, total RNA was extracted using a Total RNA Extraction Kit (Beijing Solarbio Science & Technology Co., Ltd.), according to the manufacturer’s instructions. Reverse transcription was then performed using a Universal RT-PCR Kit (M-MLV, Beijing Solarbio Science & Technology Co., Ltd). The synthesized complementary DNA was amplified by PCR over 30 cycles at 94°C for 10 s, 60°C for 10 s and 72°C for 2 kb/min using gene-specific primers listed in Supplementary Table S2. The PCR products were analyzed by electrophoresis on a 2% agarose gel, stained with ethidium bromide and visualized using a Fluor Chem HD2 system (Alpha Innotech).

For Alizarin red S and Toluidine blue staining, HUMSCs were cultured with the corresponding samples for 21 days. Alizarin red S staining was performed using an Alizarin red S staining solution (0.2%, pH 8.3, Solarbio) according to the manufacturer’s instructions. Briefly, cells were washed twice with PBS, fixed with 4% paraformaldehyde for 10–15 min, washed with deionized water, stained with Alizarin red S solution for 20–30 min, and then washed with deionized water before microscopic observation. Toluidine blue staining was performed using a cell-specific Toluidine blue staining solution (Solarbio) according to the manufacturer’s instructions. Briefly, cells were washed twice with PBS, stained with toluidine blue solution for 5 min, mixed with an equal volume of distilled water and incubated for 15 min, washed with distilled water and then observed under a microscope.

### Animal experiments

All animal work was conducted in accordance with the legislation of China and adhered to the National Guidelines for the Care and Use of Laboratory Animals. The procedures were reviewed and approved by the Institutional Animal Care and Use Committee (IACUC) of Beijing Morningrise Hi-Tech Biotechnology Co., Ltd. (No. 20230145YZE). Each animal was treated with a disposable, sterile syringe and housed individually in separate cages. The animals were fed a standard laboratory diet and kept under controlled lighting, noise and temperature.

### Surgical procedure

*In vivo* studies were carried out using 36 male SD rats weighing 360–400 g purchased from Beijing Vitonglihua Experimental Animal Technology Co., Ltd. A 1.5 cm full-thickness skin incision was made longitudinally above the medial femoral condylar under anesthesia and aseptic conditions. Then, a cavity of 2 mm in diameter and 2 mm in depth was drilled in the center of the distal femur. The experimental materials were injected into the cavity to fill the defect and then sutured. The rats were randomly divided into four groups: (i) blank, without any implantation, (ii) PMMA, (iii) BCP/PMMA and (iv) KBP. Rats were sacrificed by carbon dioxide euthanasia at 4, 8 and 12 weeks after surgery, and the leg bones were collected for micro-CT scanning and histological analysis.

### Micro-CT analysis

The distal femurs were dissected out and fixed in 4% formaldehyde. Micro-CT (Bruker, SkyScan1276, Germany) was used to scan the defect area (parameters: 10.15 µm resolution, 85 kV voltage and 200 µA current). Three-dimensional (3D) reconstruction was undergone by the manufacturer’s software. The volume of interest (VOI) was 2 mm in diameter and 2 mm in height. The bone volume fraction (BV/TV) and local BMD were calculated by the software.

### Histological analysis

New bone regeneration, bone cement degradation and inflammatory infiltrate were assessed using histological analysis. Samples were fixed in 4% paraformaldehyde for 24 h, decalcified using 10% EDTA for 5 weeks, then dehydrated in a series of ethanol concentrations (graded from 70% to 100%), and finally embedded in paraffin. Tissue sections (4 µm thick) were stained with H&E and SO/FG. These sections were then examined under a light microscope (Olympus, Japan).

### *In vivo* biocompatibility

Acute systemic toxicity was assessed following the Chinese National Standard of GB/T 16886.11-1997. Twenty healthy Kunming strain rats (weighing 35–40 g, Beijing Vital River Laboratory Animal Technology Co., Ltd.), were divided into two groups (5 males and 5 females per group). One cement extract and saline (control) were administered to the respective groups via intraperitoneal injections at a dosage of 50 mL kg^−1^. The body weights of the rats were monitored at 24 h intervals.

For the pyrogenicity test, bone cement extract (3 mL, preheated to 37°C) was administered through the tail vein to five male SD rats (weighing 230–250 g, obtained from Beijing Vital River Laboratory Animal Technology Co., Ltd.). The control group received saline. Rectal temperatures were measured before the injection and again at 24 h postinjection.

In a separate test for local reactions, dorsal subcutaneous injections of bone cement extract (0.02 mL) were administered to five Kunming rats, with another five-receiving saline as controls. Over 24 h of postinjection, observations were conducted to check for erythema, edema or induration signs. Sensitization level was determined according to Supplementary Table S3.

### Statistical analysis

All quantitative data are presented as mean ± standard deviation (SD). Comparisons were analyzed by one-way analysis of variance (ANOVA) followed by Tukey’s multiple comparisons test. A value of *P < 0.05 was considered statistically significant. *P < 0.05, **P < 0.01, ***P < 0.001.

## Supplementary Material

rbag157_Supplementary_Data

## Data Availability

All data are available upon request.
