# Supplementary material for: Human hair keratin modification enhances the biocompatibility, bioactivity and osteogenic property of BCP/PMMA bone cement
Source: Regen Biomater. 2026 Jul 22;13:rbag157. doi: 10.1093/rb/rbag157 (PMC13523852; doi:10.1093/rb/rbag157)
Supplement: Supplementary file 1 [file rbag157_supplementary_data.pdf]

Supporting Information

Human Hair Keratin Modification Enhances the Biocompatibility, Bioactivity, and Osteogenic Property of BCP/PMMA Bone Cement

Zhigang Li, Xinyu Zhao, Yuchen Cao, Yadi Niu, Haifeng Wang, Xiaoyan Gong, Peng Li, Yen Wei, and Kangmin Niu**

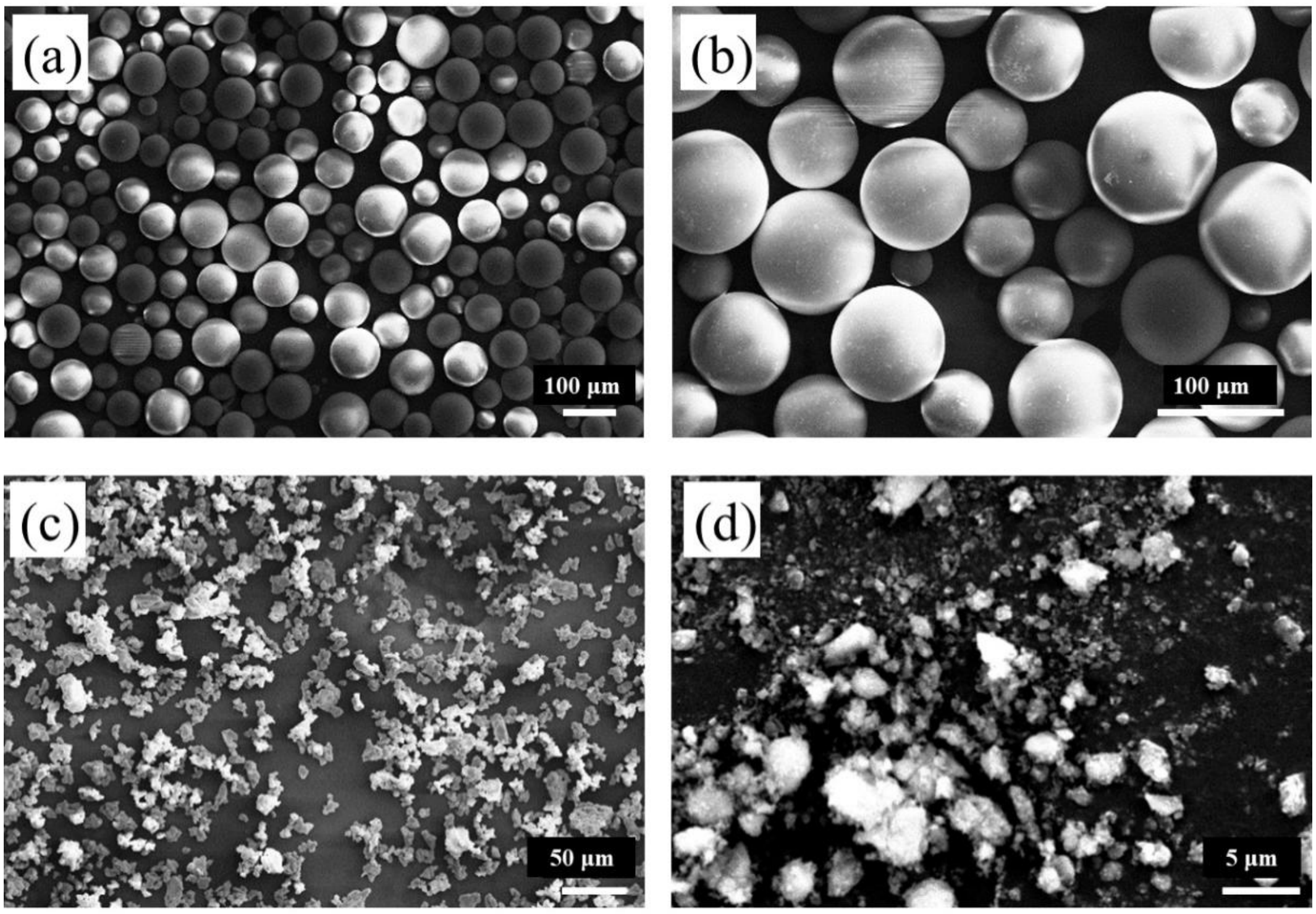

Figure S1. Scanning electronic microscope photographs of (a)(b) PMMA and (c)(d) BCP particles.

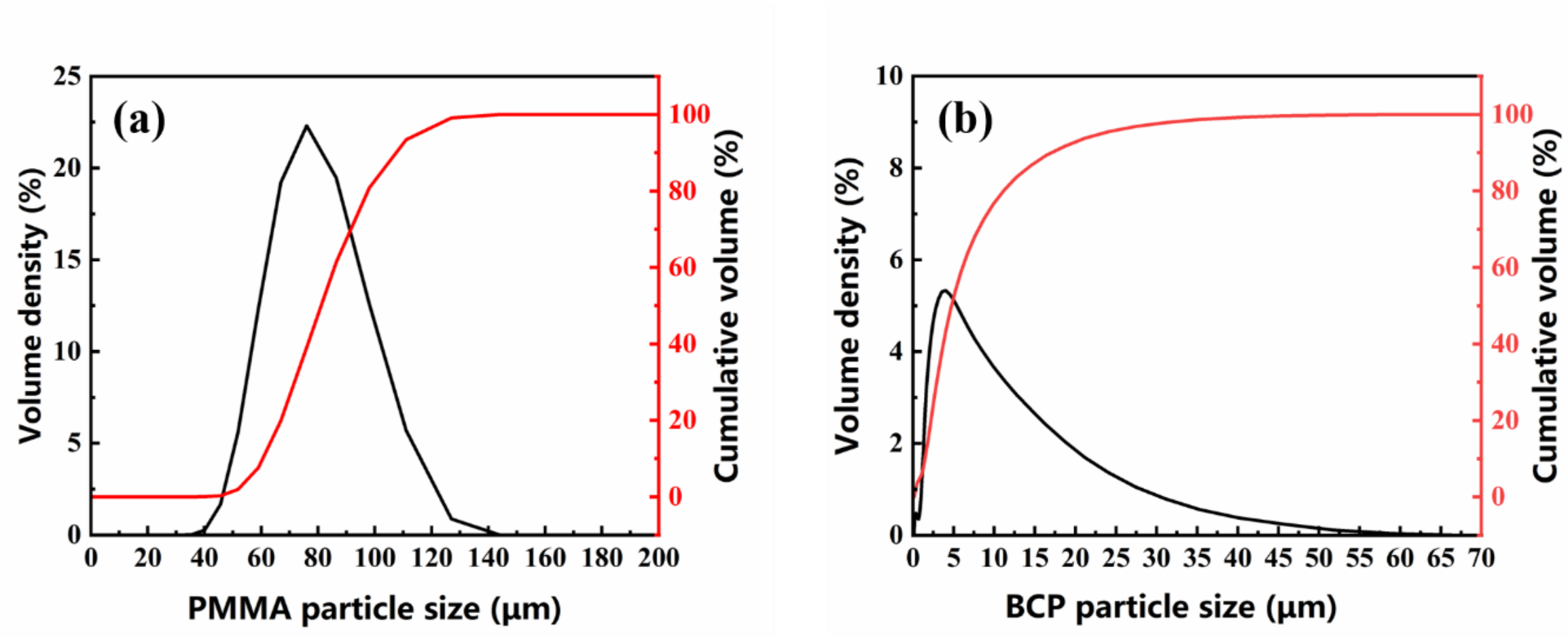

Figure S2. Particle size distribution of (a) PMMA and (b) BCP.

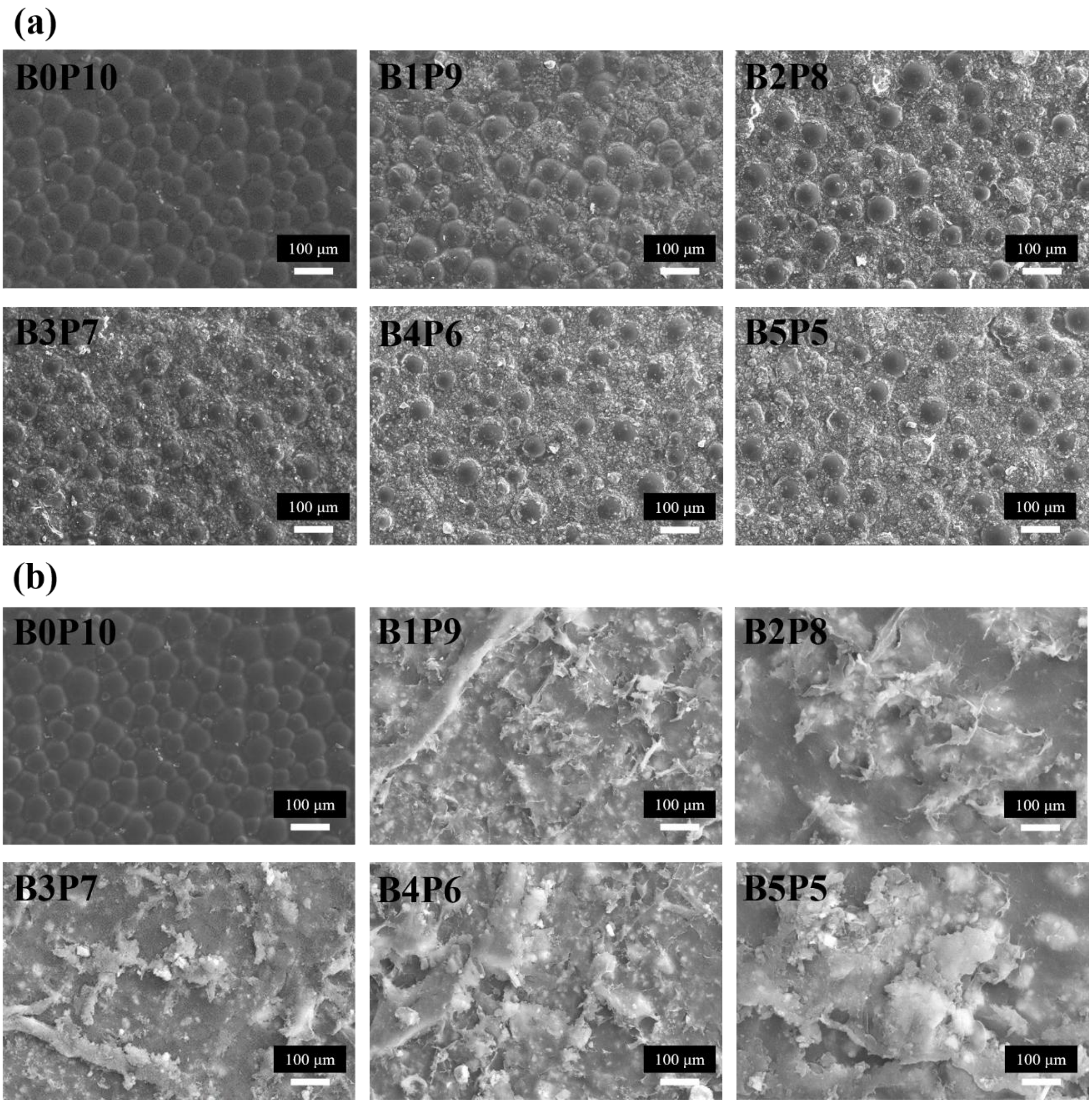

Figure S3. Scanning electronic microscope images of BCP/PMMA bone cements (a) before soaking in SBF and (b) 28 days after soaking in SBF.

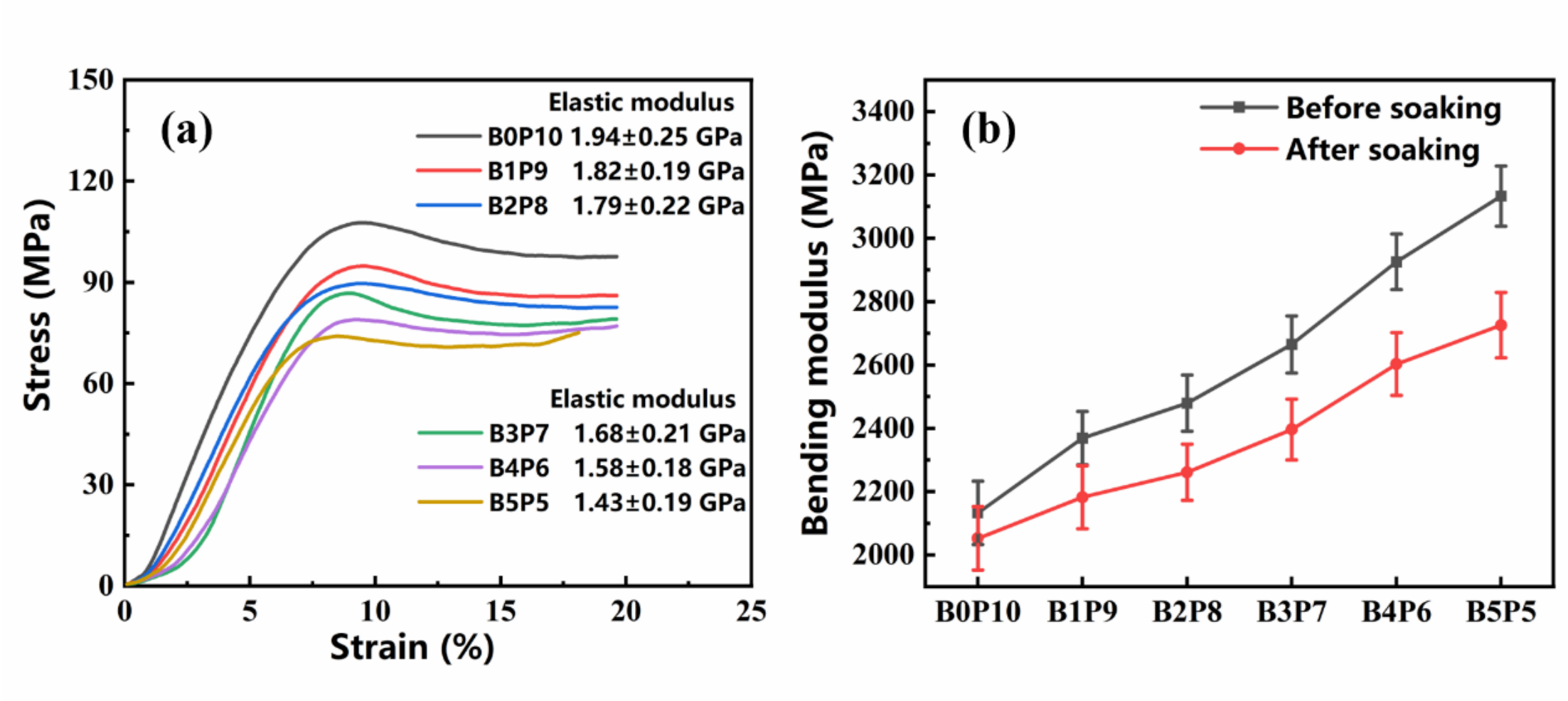

Figure S4. (a) Compressive stress–strain curves and (b) bending modulus of BCP/PMMA bone cement composites.

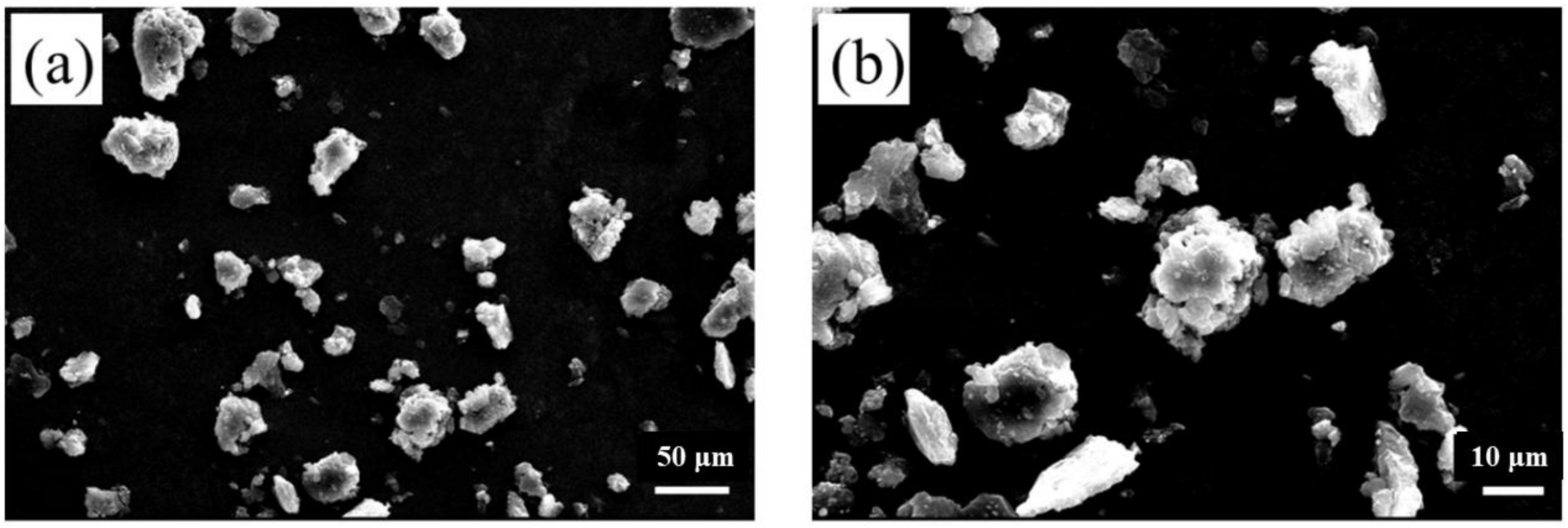

Figure S5. Scanning electronic microscope images of extracted human hair keratin powder.

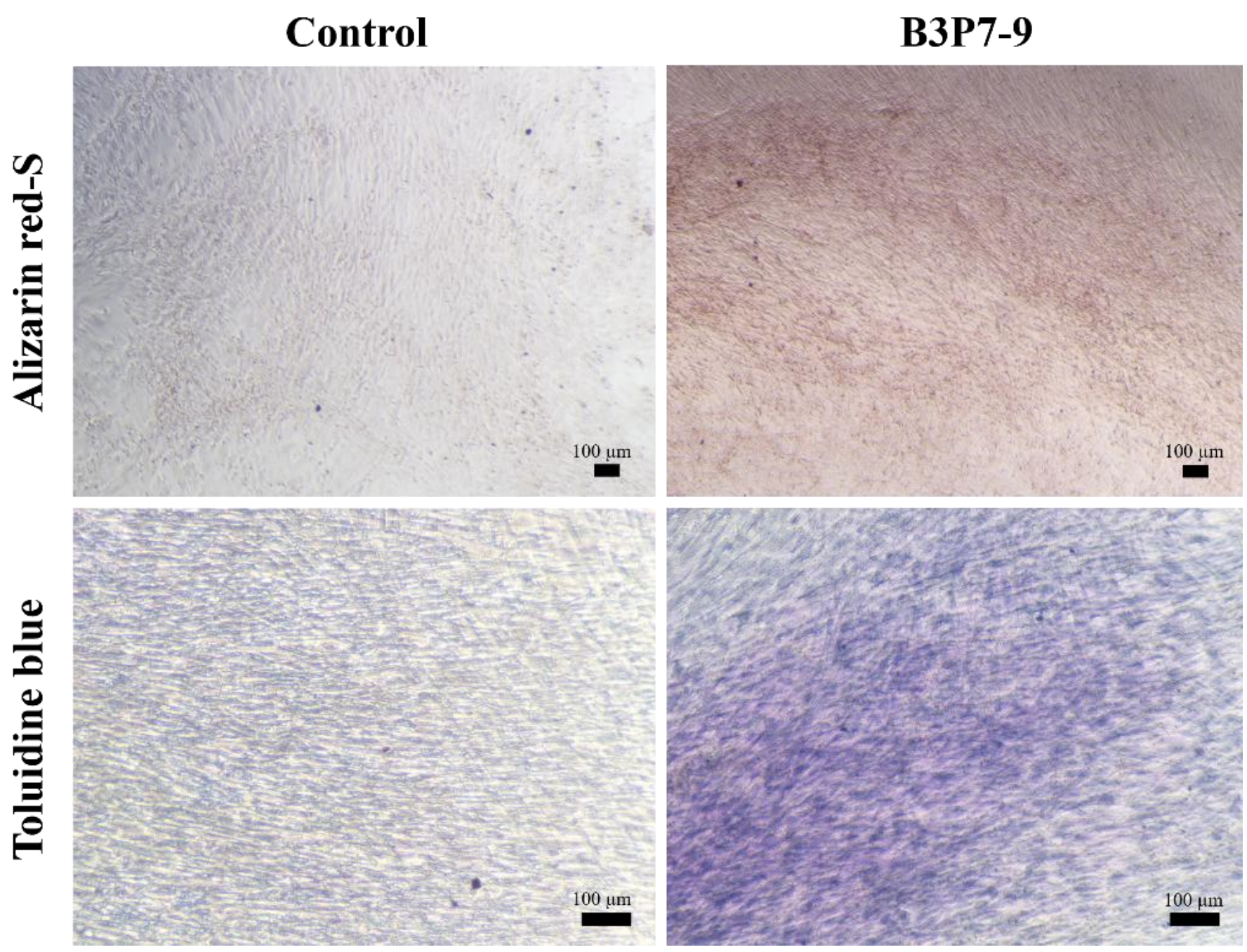

Figure S6. Alizarin red-S and Toluidine blue staining images of HUMSCs after 21 days of culture. Alizarin red-S staining indicates calcium deposition related to osteogenic differentiation, while Toluidine Blue staining indicates acidic mucopolysaccharide-rich matrix formation related to chondrogenic differentiation. Scale bar: 100 μm.

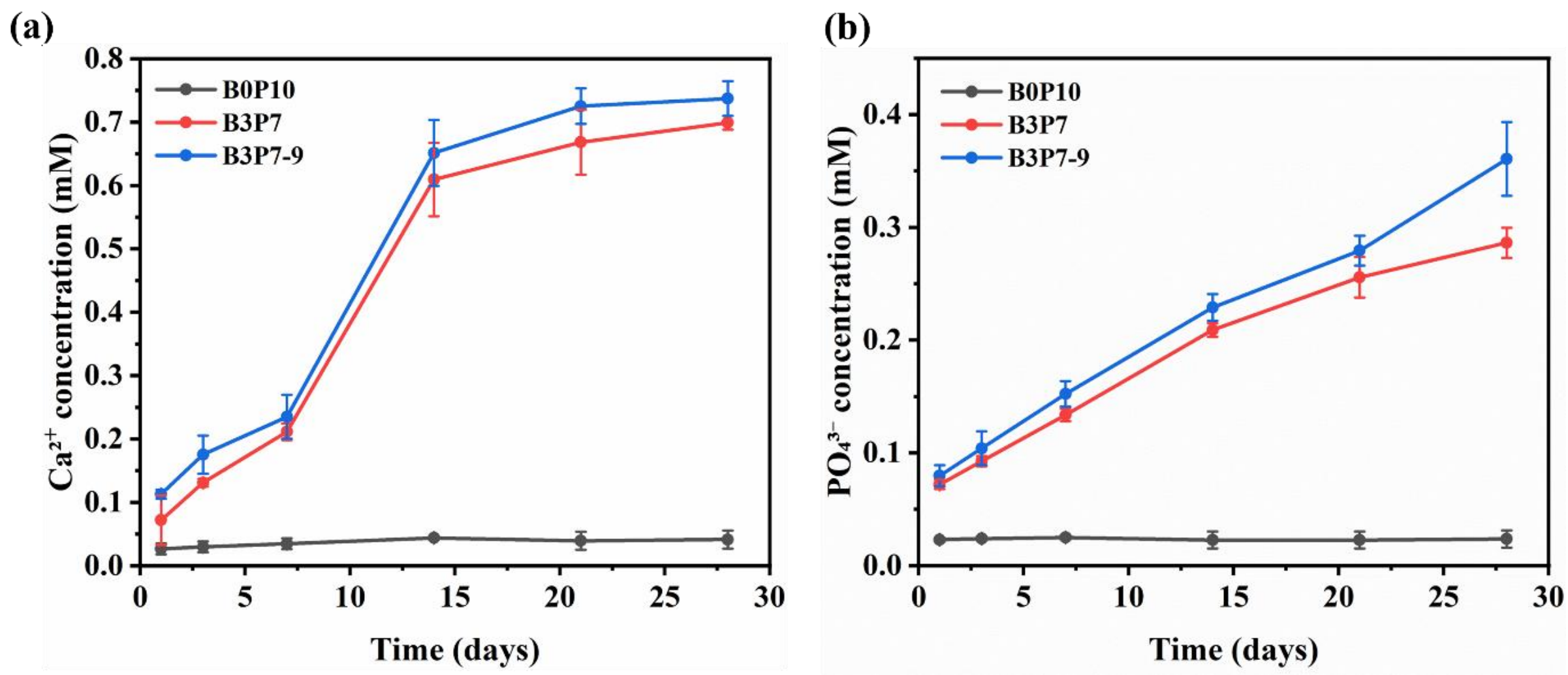

Figure S7. Calcium and phosphate ion concentrations in SBF soaking solutions during degradation. (a) Ca^{2+} concentration and (b) PO_4^{3-} concentration in the SBF soaking solutions of B0P10, B3P7, and B3P7-9 after immersion for 1, 3, 7, 14, 21, and 28 days. Data are presented as mean ± SD (n = 3).

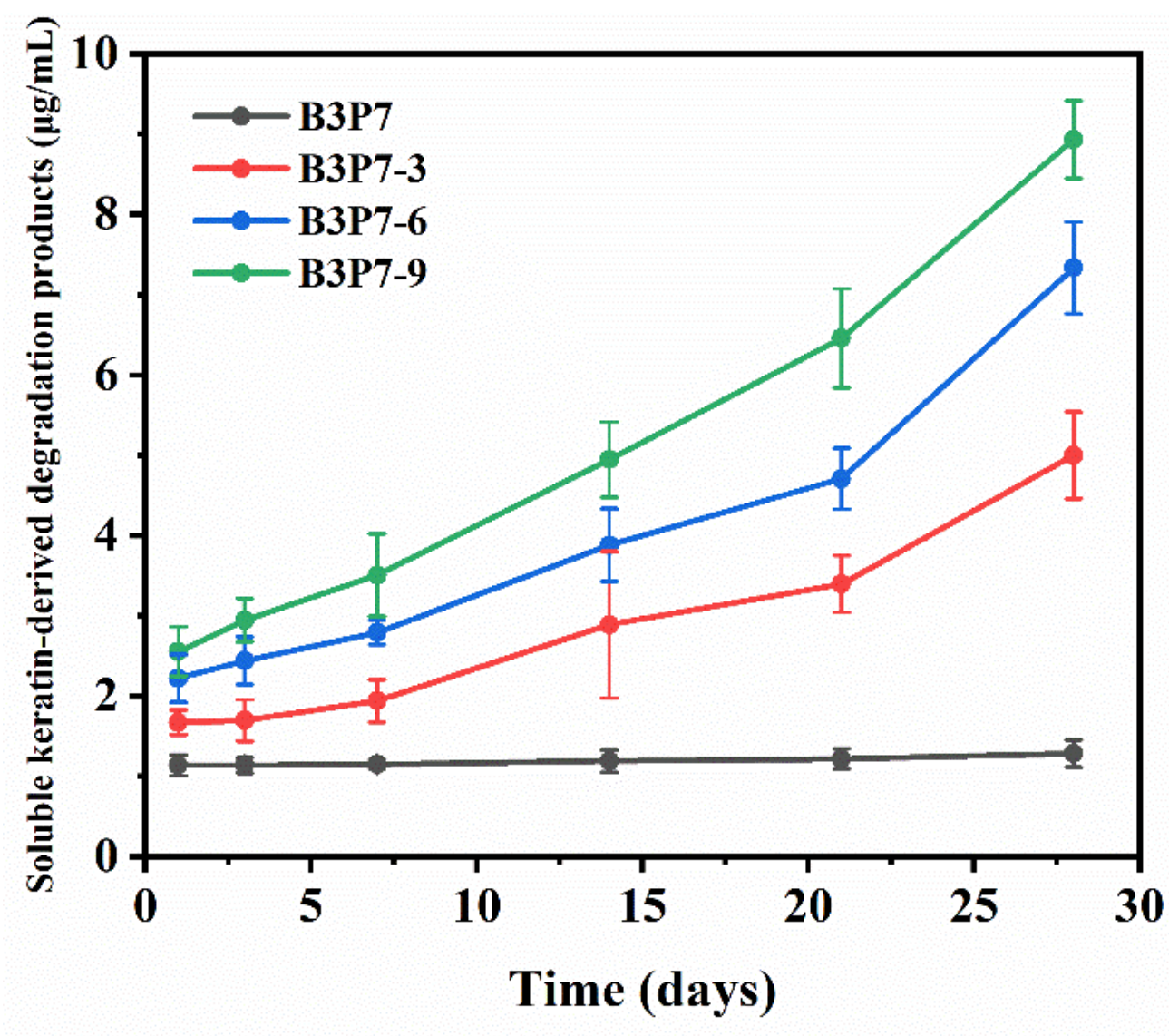

Figure S8. Release of soluble keratin-derived protein/peptide degradation products during SBF immersion. Concentrations of soluble protein/peptide in soaking solutions of B3P7, B3P7-3,

B3P7-6, and B3P7-9 after immersion for 1, 3, 7, 14, 21, and 28 days. Data are presented as mean ± SD (n = 3).

Table S1. Correspondence between cell viability and cytotoxicity level

Cell viability (%)	Cytotoxicity level	Interpretation
≥100	0	No cytotoxicity
75–99	1	No cytotoxicity
50–74	2	Mild cytotoxicity
25–49	3	Mild cytotoxicity
1–24	4	Moderate cytotoxicity
0	5	Severe cytotoxicity

Table S2. Primers used for RT-PCR

	Gene	Primer sequence (5' to 3')	Product size (bp)
Osteogenesis	Osteocalcin	AGAGCGACACCCTAGAC<br>CATGAGAGCCCTCACA	310
Chondrogenesis	COMP	CGCAGATGCTTCGGGAACTG<br>GTTGCACTCGTTGACGTCGG	302

Table S3. Determination of sensitization level

Observation	Sensitization level
No distinct change	0
Sporadic or macular erythema	1
Toxic confluent erythema	2
Severe erythema and edema	3
